# Functional signaling test identifies HER2 negative breast cancer patients who may benefit from c-Met and pan-HER combination therapy

**DOI:** 10.1186/s12964-021-00798-9

**Published:** 2022-01-08

**Authors:** Ian A. MacNeil, Salmaan A. Khan, Adrish Sen, Sajjad M. Soltani, David J. Burns, Brian F. Sullivan, Lance G. Laing

**Affiliations:** Celcuity, Inc., 16305 36th Ave N, Suite 100, Minneapolis, MN 55446 USA

**Keywords:** HER, c-Met, Combination targeted therapy, Dysfunctional signaling

## Abstract

**Background:**

Research is revealing the complex coordination between cell signaling systems as they adapt to genetic and epigenetic changes. Tools to uncover these highly complex functional linkages will play an important role in advancing more efficacious disease treatments. Current tumor cell signal transduction research is identifying coordination between receptor types, receptor families, and transduction pathways to maintain tumor cell viability despite challenging tumor microenvironment conditions.

**Methods:**

In this report, coactivated abnormal levels of signaling activity for c-Met and HER family receptors in live tumor cells were measured by a new clinical test to identify a subpopulation of breast cancer patients that could be responsive to combined targeted therapies. The CELsignia Multi-Pathway Signaling Function (CELsignia) Test uses an impedance biosensor to quantify an individual patient’s ex vivo live tumor cell signaling response in real-time to specific HER family and c-Met co-stimulation and targeted therapies.

**Results:**

The test identified breast tumors with hyperactive HER1, HER2, HER3/4, and c-Met coordinated signaling that express otherwise normal amounts of these receptors. The supporting data of the pre-clinical verification of this test included analyses of 79 breast cancer patients’ cell response to HER and c-Met agonists. The signaling results were confirmed using clinically approved matching targeted drugs, and combinations of targeted drugs in addition to correlative mouse xenograft tumor response to HER and c-Met targeted therapies.

**Conclusions:**

The results of this study demonstrated the potential benefit of a functional test for identifying a subpopulation of breast cancer patients with coordinated abnormal HER and c-Met signaling for a clinical trial testing combination targeted therapy.

Video Abstract

**Supplementary Information:**

The online version contains supplementary material available at 10.1186/s12964-021-00798-9.

## Background

The concept of coordinated c-Met (or hepatocyte growth factor receptor) and human epidermal growth factor receptor (HER) tyrosine kinase family signaling in cancer progression is well-established [1, 2]. Interaction between overexpressed HER family receptors and c-Met receptors or increased abundance of their ligands and downstream signaling constituents provide plausible resistance mechanisms to targeted therapies [3–7]. A recent in-depth review of the subject suggested suppression of HER3 with c-Met would be a likely requirement for overcoming resistance to epidermal growth factor receptor (EGFR; also known as HER1) targeted therapies [8]. Other studies have demonstrated the possibility for c-Met and HER family activation to participate in mitogen activated protein kinase (MAPK) and phosphoinositide-3-kinase (PI3K) pathway extensive interactions that drive cancer progression [9–11]. These studies and proposed coordination mechanisms have led to clinical testing of drug combinations in patients with various solid tumor types. Phase II trials with c-Met-targeting therapeutics cabozantinib and onartuzumab in combination with erlotinib showed promising results with respect to progression free survival (PFS) [12]. However, Phase III clinical trials using c-Met overexpression as an indicator for c-Met-targeted therapy and studies in which patients were randomly selected to receive combination c-Met/HER1 treatment failed to meet statistical significance required to demonstrate clinical efficacy [12]. Detection of c-Met expression level by immunohistochemical (IHC) analysis as a clinical pathology diagnostic marker has failed to accurately identify a population responsive to c-Met targeted therapies, suggesting an alternative approach is required to identify patients with dysfunctional HER and c-Met signaling who will respond to these therapies.

HER2 gene (ERBB2) amplification and/or HER2 protein overexpression is detected in approximately 15–20% of breast cancers and is associated with more aggressive disease, progression, metastasis, and poor prognosis [13–16]. Agents targeting HER2, such as trastuzumab, lapatinib, neratinib, and pertuzumab, significantly improve clinical outcomes in HER2 + patients [16, 17]. Currently, a patient’s eligibility for HER2-targeted therapies is determined using IHC or fluorescence in situ hybridization (FISH) HER2 tests [16]. However, clinical trials have indicated a weak correlation between HER2 expression or amplification levels and HER2-targeted therapy benefit [18, 19].

An individual patient’s tumor cells adaptation to genomic or proteomic aberrations and how a patient may respond to further challenges by individual or combinations of targeted therapies may be more complex than previously suggested [20–22]. The present work describes a test to define coordinated dysfunctional hypersignaling in individual patients, thus identifying the potential means to therapeutically disrupt the tumor progression with combinations of targeted therapies for HER-family and c-Met.

We previously reported the development of a novel assay to identify patients with abnormal HER2 signaling using an impedance biosensor assay measured from ex vivo cultured patient tumor cells [23, 24]. To elucidate the role of c-Met signaling and its involvement with HER family signaling as a cancer co-driver, a new test was developed based on the principle of tumor cell impedance alterations from hypersignaling as described previously for patients that we demonstrated do not have overexpressed receptors.

The test for HER2 and c-Met signaling was performed in a multiplex format known as the CELsignia Multi-Pathway Signaling Function (CELsignia) Test. The CELsignia Test measured ex vivo real-time live cell response to specific HER family and c-Met agonists to diagnose breast tumors with hyperactive HER1, HER2, HER3, HER4, and c-Met signaling activity. We report test results from breast tumor specimens obtained from 79 histopathologically HER2-negative breast cancer patients. From the resulting dataset, we estimated a c-Met signaling score cut-off to identify abnormal signaling patients and estimated the prevalence of HER2-negative breast cancer patients that have both hyperactive c-Met signaling and hyperactive signaling from at least one of the four HER-family receptors. Additionally, we report the responses to different c-Met inhibitors, HER1 inhibitor, different pan-HER inhibitors, and combinations of these therapies. We elucidate the potential synergistic involvement of c-Met signaling with HER family signaling and we report comparison data to traditional markers of cell stress that would be impractical to apply in a clinical setting. Finally, we verified the utility of the dysfunctional signaling test in a mouse xenograft model.

## Methods

### Chemicals and reagents

Recombinant human epidermal growth factor (EGF) and neuregulin 1b (NRG1b) were purchased from R&D Systems (Minneapolis, MN). Recombinant human hepatocyte growth factor (HGF) was purchased from Peprotech (Rocky Hill, NJ). Collagen was obtained from Advanced Biomatrix (Carlsbad, CA) and fibronectin was obtained from Sigma (St. Louis, MO). Neratinib, erlotinib, tepotinib were purchased from SelleckChem (Houston, TX) and prepared at stock concentrations in fresh 100% DMSO (Amresco) before final dilution into assay medium. The monoclonal anti-HER2 antibody, 2C4, was expressed and purified from a mouse hybridoma cell line obtained from ATCC (Manassas, VA). Drugs for in vivo studies were prepared in 10% captisol.

### Tissue and cell specimens

HER2-negative tumor specimens were obtained from excess, de-identified, surgically resected human breast cancer tissue. Tissues were obtained from multiple clinical sites located across the United States. IRB exemption was granted by Liberty IRB (Columbia, MD) after determining that the proposed research did not involve human subjects as defined under 45 CFR 46.102(f); Liberty IRB has full accreditation with the Association for the Accreditation of Human Research Protection Programs (AAHRPP). For inclusion in this study, each specimen was required to meet the following criteria: (1) a minimum specimen weight of 10–20 mg; (2) derived from a confirmed breast cancer (any stage, including recurrence) patient with identifiable tumor mass; and (3) obtained from female patients > 18 years old. The breast tumor samples were confirmed for no HER2 amplification using clinical standard procedures by the accredited clinical pathology lab of the institutions supplying the specimen. Additional File 1: Table S1 summarizes the patient characteristics based on age, stage of cancer, tumor histology, and expression of estrogen receptor on tumor cells.

### Tumor cell culture

HCC1954 cells were obtained from ATCC (Manassas, VA). The cells were cultured and maintained in the laboratory in RPMI medium (Corning; Tewksbury, MA) supplemented with 10% FBS (Hyclone, Marlborough, MA) and l-glutamine (Sigma; St. Louis, MO) according to ATCC specifications [24]. Tissue extraction from tumor specimens and primary cell culture was carried out as described previously [24, 25]. Briefly tissue was minced to less than 2 mm length, enzymatically digested, and plated onto collagen-fibronectin coated 4-well culture plates where cells were typically cultured on average for less than fourteen days before transfer to the analytical test procedure.

### Flow cytometry

HER2 receptor expression data was confirmed by flow cytometry analysis. HER2 and c-Met expression was evaluated in primary cells isolated from the tumors at the time of CELsignia testing, using anti-HER2-PE (BioLegend; San Diego, CA) and anti-HGF-c-Met-Ax488 (R&D Systems; Minneapolis, MN) antibodies (Table S2). Fluorescence data was captured on a BD FACSCalibur (BD Biosciences; San Jose, CA) equipped with a 488-nm and 637-nm laser and data analysis was done using the FlowJo 2 software (FlowJo LLC; Ashland, OR).

For analysis of cell toxicity, single cell suspensions were stained with TMRE (200 nM for 30 min), washed with FACS buffer, and stained with Annexin V-AlexaFluor647 (Biolegend; San Diego, CA) following the manufacturers’ instructions. The TMRE/Annexin V stained cells were resuspended in FACS buffer containing Sytox Blue (Thermo Fisher; Waltham, MA) and analyzed on a Novocyte 3000 FACS instrument and NovoExpress software (Agilent; Santa Clara, CA). For analysis of intracellular phosphoproteins by FACS, cells were stained using a Live/Dead fixable dye (Zombie NIR; BioLegend), fixed using 1.6% paraformaldehyde, permeabilized with cold-methanol, and then stained using antibodies towards AKT-pS473 (Cell Signaling Technology; Danvers, MA) and FAK-pS910 (BD Biosciences) (Table S2) prior to FACS analysis.

### CELsignia real-time live cell testing with agonists and antagonists

Zero passaged cells were counted using an NC-250 and seeded into collagen-fibronectin coated 96-well E-plates and prepared for analysis on an Agilent xCELLigence RTCA instrument. Real-time live cell responses to specific HER3, HER1, and c-Met agonists (3 nM NRG1b, 0.3 nM EGF, 0.08 nM HGF, respectively at 18 h post drug treatment) alone and in combination with and without antagonists were measured and quantified using an xCELLigence RTCA impedance biosensor (Agilent) as described previously [24]. Cells were activated with EGF, NRG1b, or HGF at approximately the EC90 determined from titrations of at least 20 patients. Following 18 h of antagonist treatment, cells were treated with indicated amounts of receptor agonists and impedance changes were recorded for an additional 4 h. Impedance data analysis was performed using TraceDrawer (Ridgeview Instruments AB, Uppsala, Sweden) to derive reported values in 4 h signaling units.

### CELsignia test score

For the signaling function test, the 96-well biosensor E-plate was set up as follows: Test wells included:

- C (patient cells only with control media)
- CF1 (Patient cells + pathway factor NRG1b)
- CDF1 (Patient cells + anti-HER2 dimerizing antibody + pathway factor NRG1b)
- CF2 (Patient cells + pathway factor EGF)
- CDF2 (Patient cells + defining anti-HER2 antibody + pathway factor EGF)
- CF3 (Patient cells + pathway factor HGF)

HER2 signaling score was calculated as described previously where differences between the CF and CDF, indicating loss of HER2 dimer contribution (assessed from applying HER2 binding, dimer blocking antibody) to HER1 and HER3 ligands, were applied to determine homodimer signaling from HER2 involved heterodimer signaling. HGF activated c-Met signaling, the c-Met signaling score, was defined as CF3-C using the equation below:

$$cMet\;signaling\;score = \mathop \sum \limits_{i = 0}^{240} \left( {CF3i - Ci} \right)$$

where variables are defined as: *i* = steps for each minute the cell attachment signal is recorded during the test. C_i_ = Control, no perturbation factor added to test cells. CF3_i_ = Cells with HGF (c-Met pathway factor).

**Fig. 1:**
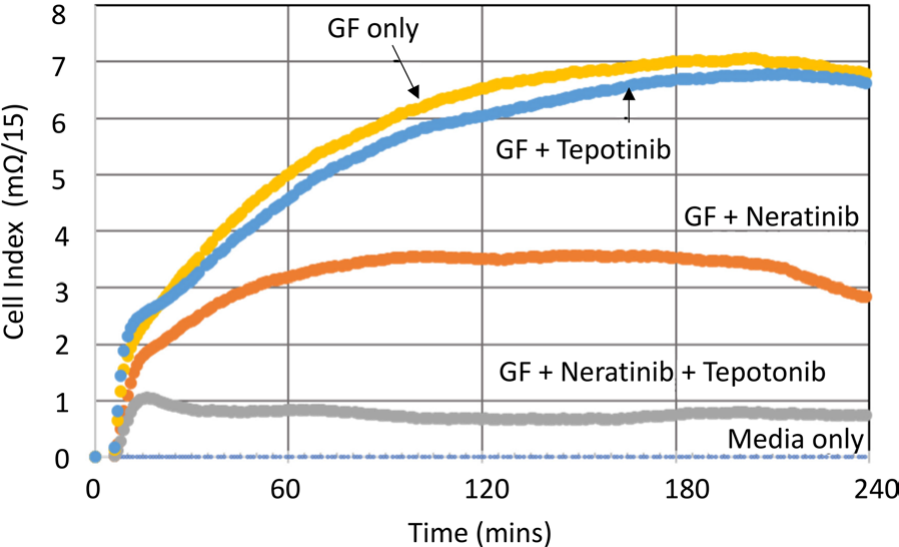
Combination of pan-HER and c-Met inhibitors effectively block the HER-family and the c-Met receptor coactivated functions. Data for an example patient, C135, demonstrates the efficacy of combining neratinib and tepotinib against abnormal stimulation response generated by simultaneous application of the three growth factors (GF)—NRG, EGF, and HGF. Each curve is labeled in the figure to indicate the inhibitor(s) that was added to different wells containing the same number of cells. The order of efficacy response compared to the growth factor only well (at the top of the graph) from low response to high is as follows—Tepotinib alone is least efficacious, Neratinib alone is more efficacious, and the most efficacious treatment is the combination of neratinib and tepotinib data curve that in the figure is just above the dotted blue baseline for untreated cells (no agonist, no antagonist control)

In brief, the CELsignia test score is calculated by summing the individual minute readings over a 240-min time period where each reading reflects the change in impedance signal at that time point (units of Cell Index, simple function of milli Ohms) compared to the same time point of a well containing cells with control media only (Fig. 1).

### Determining IC_50_ values

Owing to limited numbers of non-passaged or low passaged primary cells, 1000-fold, five-point dose response for antagonists to HER-family and c-Met receptors was performed for each corresponding agonist stimulus (i.e. NRG1b/EGF for HER-family activation and HGF for c-Met activation) in a live-cell CELsignia assay and the observed values were averaged. Drug additions were made 18 h prior to agonist addition. The measurand recording period of four hours began after the agonist addition. Response was plotted as a percent of the maximum response to the agonist observed in the absence of any antagonist using GraphPad Prism software and IC_50_ values were derived using non-linear regression.

### Data analysis

The distribution of HER2 signaling scores from the 79-patient data set was first evaluated for compatibility with the distribution of HER2 signaling scores obtained from a previous study of 114 patient samples test (24) using the Kolmogorov–Smirnov two-sample test for identity of distributions with respect to HER2 signaling scores. The test statistics obtained was D = 0.10256, with a *p* value of 0.7146, indicating that there was no significant difference between the HER2 signaling scores of these two study groups.

The c-Met signaling scores for the 79-patient data set were analyzed to determine whether the population was comprised of a single group or multiple sub-groups of patients, using the normal mix EM procedure in the R package “mixtools’ to fit a normal mixture model. Two and three component fit runs were subsequently performed, along with a baseline single-component model.

**Fig. 2:**
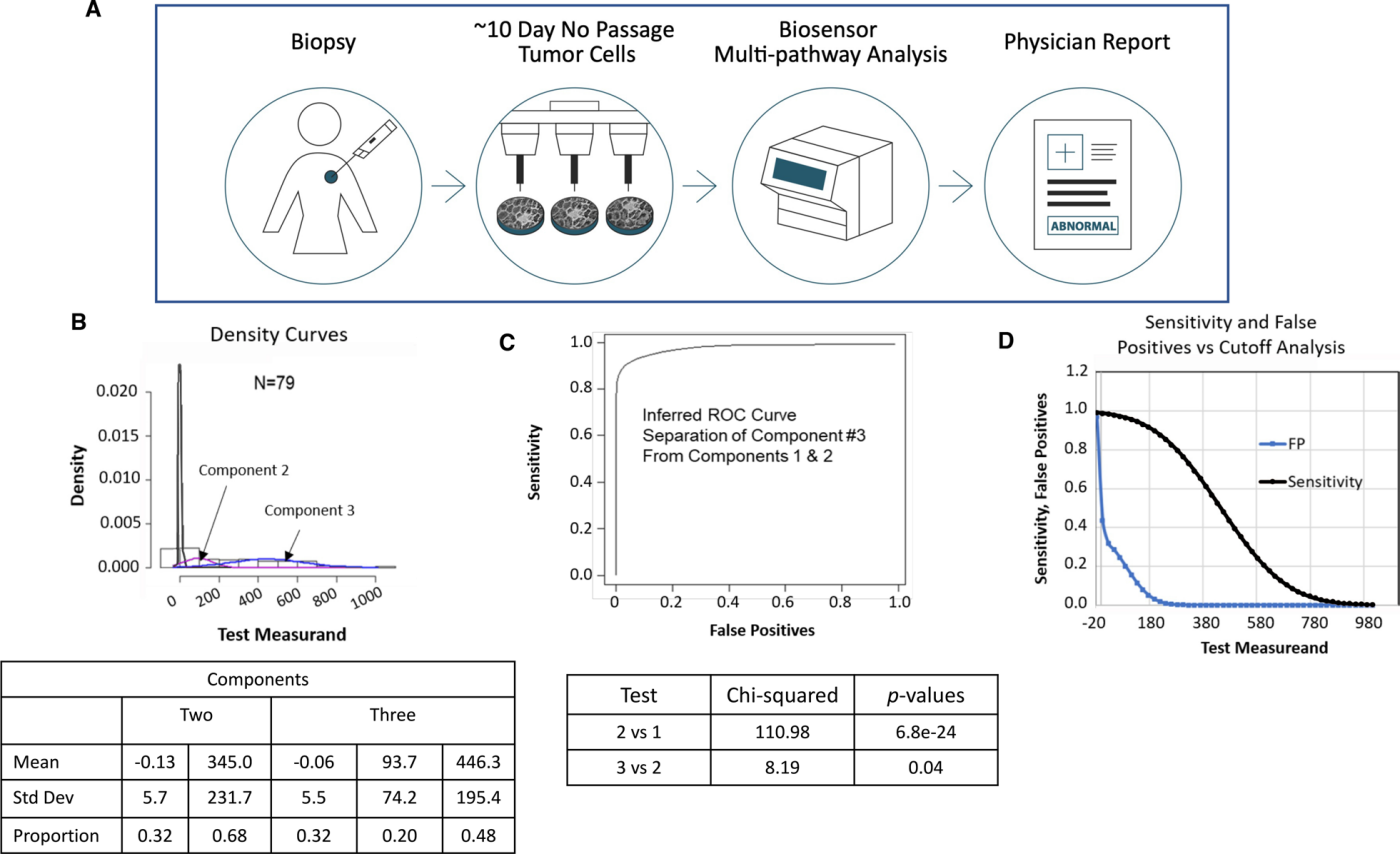
Clinical test procedure and c-Met cutoff determination and prevalence analysis using the CELsignia test in a population of HER2 negative breast cancer patients. **a** Clinical test procedure summary: A 14-gauge to 18-gauge needle biopsy containing about 25 mg of tumor tissue is removed and sent by overnight courier to the Celcuity lab in Minneapolis. After a brief, no passage, culture establishes the viability of the tumor cells, a CELsignia multipathway test is performed using an impedance-based biosensor (96-well format device). Typically, an abnormal signaling report is returned to the requesting physician in less than 14 days. **b** CELsigniaTest Score distribution density analysis: The likelihood ratio test for the number of components gave the following parameters. For the three-component model, the components 2 (purple) & 3 (blue) means are more than four standard deviations apart. **c** ROC curve for the three-component fit plots sensitivity versus false positive rate for various cut-off points. Formal significance testing shows that a two-component mixture fits much better than a common normal distribution, and a three-component mixture fits better than two. **d** False positive rate (blue line) and sensitivity (black line) are presented as a function of the cutoff used

The two-component fit separates the bottom one-third of patients from the top two-thirds of the distribution. The three-component fit essentially separates the upper population of the two-component fit into a smaller intermediate population and a larger upper population. The three-component fit superimposed on a histogram of the data is shown in Fig. 2b. There is overwhelming support for more than one population. The evidence for three populations rather than two is nominally significant as shown in the formal likelihood ratio tests (*p* = 0.04; Fig. 2c). The three-component fit splits the population roughly in half, the 48% from component 3 and the 52% from the composite of components 1 and 2. The right-most fitted component comprises 48% of the population and has a mean and standard deviation of 446 and 195 respectively (Fig. 2b).

For the three-component fit, we plotted sensitivity versus the false positive rate for different cutoff points in a receiver operator characteristic curve (ROC; Fig. 2c). Each point on the ROC curve represents a sensitivity/false positive pair corresponding to a particular cutoff value. Figure 2c is a graphic depiction of the numbers underlying the ROC curve for the sensitivity and false positive rates.

### Agonist and antagonist interactions defined by median effects

The classical median effects method of Chou and Talalay was employed to define agonist and antagonist interactions [26]. Agonist EC50 and antagonist IC50 were determined from their CELsignia test scores as single perturbants or matched agonist/antagonist. The EC50 or IC50 concentrations were used to set the reagent ratios for performing paired component median effects titration testing by CELsignia methods. A combination of two or more of the CELsignia test concentrations of EGF, NRG, and HGF showed signs of some form of feedback that was further reinforced by median effects analysis, demonstrating antagonism between growth factors. Upon finding the combination of agonists were surprisingly antagonistic towards each other by CELsignia data, we selected the agonist combination of EGF and HGF at a concentration ratio below the EC50 that gave a strong CELsignia test signal in an approximately additive manner for the next step of antagonist (drug) median effects combination testing (Additional File 1: Fig. S1). The two inhibitors (neratinib and tepotinib) were paired at IC50 concentrations as determined from the impedance test analysis of the single growth factor (Tepotinib + HGF has tepotinib IC50 = 7 nM and neratinib + EGF has neratinib IC50 = 15 nM), and thus a 1:2 tepotinib to neratinib ratio was selected for the median effects treatments. Dose escalations of combined drugs were tested at the paired inhibitor ratios on combined EGF and HGF stimulations according to the method of Chou and Talalay.

### Statistical analyses

c-Met signaling activity in the CELsignia test was defined as the difference between the HGF growth factor signal and the untreated control cell signal measured over a four-hour period. The dataset of c-Met signaling scores obtained from the CELsignia test of tumor specimens from 79 HER2-negative breast cancer patients was first analyzed to determine whether the population was comprised of a single group or multiple sub-groups of patients. This analysis used the normalmixEM procedure in the R package “mixtools’ to fit a normal mixture model and generate ROC curves. Two and three component fit runs were subsequently performed along with a baseline single-component model. Sensitivity and specificity rates were derived using the R package with the following code:

$$\begin{aligned} & {\text{mix}}\left[ {{1}:{2}} \right] < - {\text{mix}}\left[ {{1}:{2}} \right]/{\text{sum}}\left( {{\text{mix}}\left[ {{1}:{2}} \right]} \right) \\ & {\text{FP}} < - {\text{mix}}\left[ {1} \right]*{\text{pnorm}}\left( {\left( {{\text{mus}}\left[ {1} \right] - {\text{cutter}}} \right)/{\text{sds}}\left[ {1} \right]} \right) \\ & \quad \quad + {\text{mix}}\left[ {2} \right]*{\text{pnorm}}\left( {\left( {{\text{mus}}\left[ {2} \right] - {\text{cutter}}} \right)/{\text{sds}}\left[ {2} \right]} \right) \\ & {\text{sens}} < - {\text{pnorm}}\left( {\left( {{\text{mus}}\left[ {3} \right] - {\text{cutter}}} \right)/{\text{sds}}\left[ {3} \right]} \right) \\ \end{aligned}$$

where ‘cutter’ is the cutoff, ‘mus’ are the means of the three components, ‘sds’ are the standard deviations of the three components, and ‘mix’ is the relative proportion of the first and second components in the three-component mixture.

#### Xenograft study

A selection of an appropriate cell line was necessary owing greatly to the limited commercially available tumorigenic breast cancer mouse models with the abnormal signaling function of interest to this study. Female NSG (NOD SCID gamma; NOD.Cg-*Prkdc*^*scid*^* Il2rg*^*tm1Wjl*^*/*SzJ) mice were procured from Jackson Labs (Bar Harbor, Maine, USA). The mice were injected in their fat pad with HCC1954 cells dissolved in 150 µL of a 50% Matrigel solution. Tumors were allowed to form until they reached a minimum average size of 150 mm^3^. At this time, the tumor bearing cohort was divided up into 10-mouse treatment groups of uniform distribution of tumor sizes, just prior to initiating treatment. Tumor size and body weights were measured a minimum of twice per week. Upon completion of the study period, the mice were euthanized for necropsy and sample/tissue collection at the study endpoint or when tumor size reached ≥ 2 cm^3^.

## Results

### Her2 and c-Met receptor are not overexpressed on test tumor cells

**Fig. 3:**
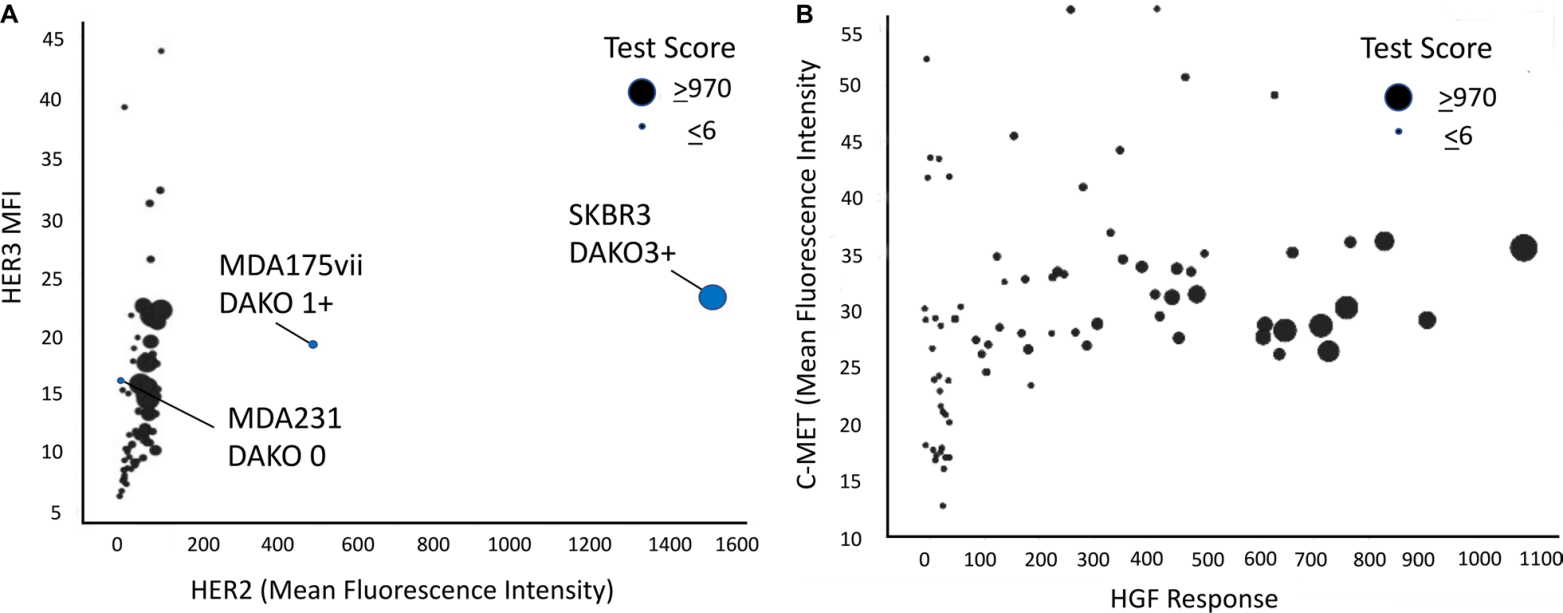
HER2 and c-Met receptor are not overexpressed on test tumor cells. **a** Flow cytometry analysis of HER2/HER3 expression confirmed the HER2- status designated by IHC and/or ISH. Seventy-nine patient samples were analyzed for HER2 and HER3 expression using flow cytometry as described in the methods. The mean fluorescence intensity (MFI) of the samples were compared to three cell lines used as standards in the DAKO HercepTest. (SKBR3-DAKO 3+, MDA175vii- DAKO 1+, MDA231-DAKO 0). Size of the circles indicates the HER2 signaling CELsignia score. **b** Comparison of expression levels of HGF receptor (c-Met) to HGF response on 79 patient samples using flow cytometry as described in the methods. The size of the circles in both panels indicates the HER2 signaling CELsignia score

We confirmed clinical pathology reports for each patient where the normal expression level of HER2 receptors was a selection criteria for enrollment in this study. We also quantified the expression of c-Met receptors on cells obtained from the same patient tumors. For both confirmations, flow cytometry analysis was performed using antibodies targeting Her2, Her3 and c-Met (Fig. 3a, b). Flow cytometry results were concordant with the standard clinical IHC test evaluations for HER2 that were provided for each specimen by the clinics. Expression of c-Met did not correlate with hepatocyte growth factor (HGF) response in the CELsignia test.

### CELsignia multi-pathway signaling function test

Figure 2A outlines the clinical test procedure workflow, including a 25 mg biopsy from a patient, a tissue processing step to isolate the tumor cells, the test analytical phase with a biosensor readout, and the actionable report for the physician of target dysfunctional signaling for the patient. Briefly, as described previously [23], we obtained live tumor specimen from which a heterogenous population of tumor cells was prepared for a live cell test. The CELsignia test uses an impedance change measurement activated by ligand addition to live tumor cells adhered specifically to microelectrodes located within the bottom of a 96-well plate. More descriptive detail of the test can be found in the Methods section.

For the present study, we obtained 79 HER2-negative breast cancer patient tumor specimens for evaluation using the CELsignia test. The main goal of the study was to identify c-Met and HER-family hypersignaling in these patient tumors (Table S1). The CELsignia test was performed on cell samples cultured from each patient tumor specimen to evaluate HER-family and c-Met signaling activity as described previously [24], applying EGF, NRG, and HGF agonists. Each of the wells containing a patient’s tumor cells in a 96-well biosensor plate was individually recorded for each patient as described in the Methods section.

We confirmed that the HER family signals arising from EGF and Neuregulin (NRG) agonism for this set of 79 patients were consistent with previous studies [23].

*Cutoff Determination for Abnormal c-Met Signal Function:* The c-Met signaling scores for the 79-patient data set were analyzed using mixtools in the R statistical package and determined to be comprised of multiple sub-groups of patients, with a three-component fit best describing the population test score distribution. The highest value fitted component comprising 48% of the population, had a mean of 446 and a standard deviation of 195 (Fig. 2b).

For the three-component fit, we plotted sensitivity versus the false positive rate for different cutoff points in an inferred receiver operator characteristic curve (ROC; Fig. 2C). Each point on the ROC curve represents a sensitivity/false positive pair corresponding to a particular cutoff value. Figure 2d is a graphic depiction of the numbers underlying the ROC curve for the sensitivity and false positive rates. At a cut-off value of 250 signaling units for HGF agonism, the test specificity is > 99% (FP < 1%) with a test sensitivity of 84%, indicative of a highly useful clinical test. The CELsignia test identified 19 of 79 HER2-negative patient samples (24.1%; 95% CI = 16–32%) with both hyperactive c-Met signaling and at least one hyperactive HER-family receptor signaling (Additional File 1: Fig. S2).

### Determining the IC_50_ values of pan-HER inhibitors against NRG1b and EGF agonist combination in a real-time live cell assay

**Fig. 4:**
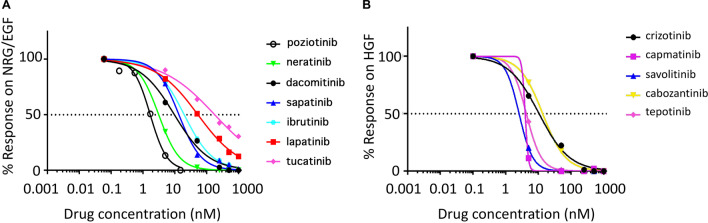
Determining the IC_50_ values of pan-HER and c-MET inhibitors from a patient-derived sample (Patient R66) **a** Percent response of stimulation of NRG1b/EGF signal (two-growth factor cocktail) when treated with a 5-point, 1000-fold titration of the six indicated pan-HER antagonists, exponential log-scale fit with GraphPad Prism. **b** Percent response of stimulation of HGF signal when treated with a 5-point, 1000-fold dose response of the five indicated c-Met antagonists

Having identified 19 tumor specimens with > 99% test specificity for hyperactive HER and c-Met signaling, we next wanted to determine the potency of the pan-HER inhibitors for HER initiated dysfunctional signaling on these breast cancer specimens. Cells isolated from patient tumors exhibiting hyperactive HER and c-Met signaling were treated with a panel of six HER receptor antagonists. Using the limited number of low passage primary tumor cells available, an IC_50_ value of each antagonist was determined using a five-point 1000-fold titration assay described in the Methods. A representative dose–response curve of cells from patient R66 to stimulation with NRG1b/EGF following pre-treatment with the indicated antagonists is shown in Fig. 4a. A total of six primary cell lines isolated from as many patients were included in this IC_50_ titration test and the average values are summarized in Table 1. The IC_50_ values for pan-HER antagonists against NRG1b/EGF stimulation determined from our real-time live cell assay align with what was previously reported in cell-free assays.

**Table 1:** IC_50_ of pan-HER inhibitors applied before stimulation with HER agonists from six primary breast cancer cell samples with hyperactive HER- and c-Met signaling

Pan-HER inhibitors	Average IC_50_ (nM)
Poziotinib*	1.23
Neratinib	4.81
Ibrutinib	13.10
Dacomitinib	22.06
Sapitinib	41.28
Lapatinib	137.27
Tucatinib^#^	333

*Poziotinib average IC_50_ was derived from dose response on three primary breast cancer cell samples

^#^Tucatinib (HER2 specific) average IC_50_ was derived from dose response on five primary breast cancer cell samples

### Determining IC_50_ values of c-Met inhibitors against HGF agonist in a real-time live cell assay

The IC_50_ value of c-Met receptor antagonists were determined in a similar fashion as described above for the Her2-receptor antagonists. Six primary cell lines were treated with c-Met receptor antagonists prior to stimulation with HGF. The dose response curve for each of the six antagonists over a 1000-fold dilution was plotted and the IC_50_ values were determined by averaging the values obtained from all the responsive cell lines (Table 2). Figure 4b shows representative dose response curves for one patient for the c-Met receptor antagonists following HGF stimulation. As observed in the case of Her2 signaling, the IC_50_s for c-Met antagonists against the HGF response determined from real-time live cell assay align with what is reported in cell-free assays (https://www.selleckchem.com/c-Met.html).

**Table 2:** IC_50_ of c-Met inhibitors applied prior to HGF stimulation in the CELsignia test for six primary breast cancer cells with hyperactive both ErbB- and c-Met signaling

c-Met inhibitors	Average IC_50_ (nM)
Capmatinib	3.10
Savolitinib	3.56
Tepotinib	14.70
Cabozantinib	27.36
Crizotinib	28.21

### Cross-talk analysis of individual antagonists against EGF, NRG1b, and c-Met illustrates receptor signaling adaptation

**Fig. 5:**
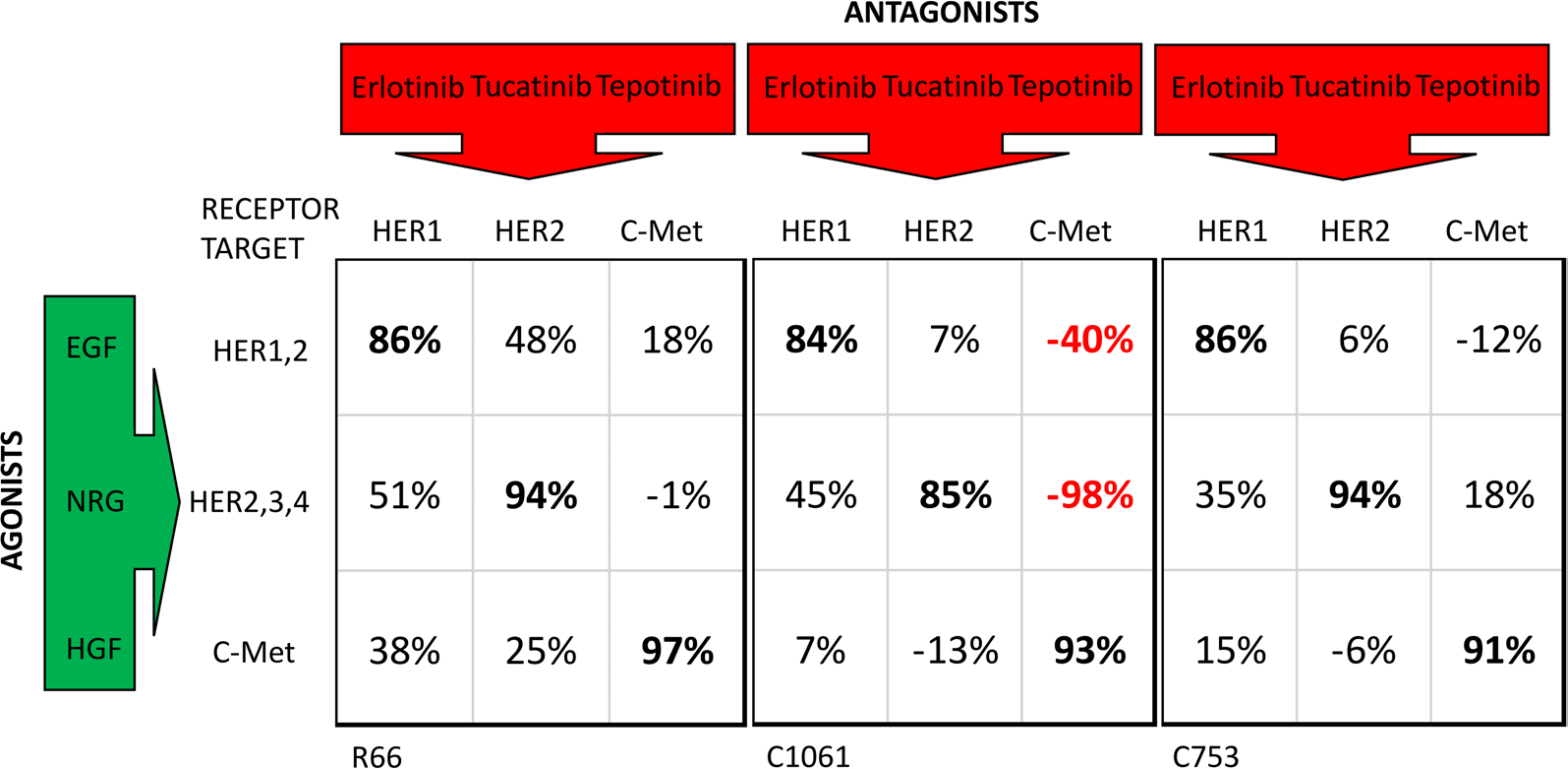
Cross-talk analysis of individual antagonists against EGF, NRG1b, and c-Met illustrates receptor signaling co-involvement. Percent inhibitions of abnormal signaling are listed for paired antagonists (Tables 5, 6, 7) . The data in Fig. 5 demonstrates for three different HER2 negative patients where treatment with the antagonist drug indicated at the column head affects the signaling generated by the agonist indicated at the matching row for each patient. For ‘on target’ pairing of drug and agonist for each patient shown, the data for duplicate wells demonstrate > 85% efficacy at reducing the abnormal signaling (indicated in bold font) directly related to the target binding receptor indicated in column headings. When considering ‘off target’ effects for patient C1061 for example, Tepotinib treatment leads to a 98% increase in signaling (negative value for inhibition, in red font) upon NRG addition and 40% increase for EGF addition. For C1061 and patient C753, treatment with a HER2 specific antagonist leads to an increase in HGF signaling

The CELsignia test was next used to determine the extent of signaling adaptation to drug targeting one receptor type occurring with signal initiation at a different target receptor. Primary tumor cells derived from three HER2-negative exemplary breast cancer patients were treated with combinations of agonists and inhibitors for HER1 (Erlotinib; 500 nM), HER2 (Tucatinib; 250 nM), or c-Met (Tepotinib; 50 nM). A 3 × 3 grid of different wells each containing the patient cells treated with one of the inhibitors were then individually stimulated with agonists EGF (0.3 nM), NRG1b (3 nM), or HGF (50 pM). Percent-inhibition of each growth factor signal over a four-hour period by the three antagonists applied individually was determined and is illustrated in Fig. 5. The data reveals the inhibition specificity of the drugs as well as some activity on other pathways initiated by unmatched agonists. A negative value for percent inhibition indicated an enhanced impedance response to the corresponding growth factor stimulation, interpreted to mean increased signaling. For example, the drug activation response (negative percent inhibition) on HER1-4 for patient C1061 upon tepotinib treatment indicates receptor activation perhaps by release of negative feedback and provides evidence for reduced efficacy of the tepotinib single drug treatment approach. Additionally, for patient C1061, the larger increase in HER2-4 signaling would explain why a HER1 targeted therapy such as erlotinib would also be ineffective, even if combined with a c-Met targeted drug.

### Selection of HCC1954 cell for CELsignia in depth analysis of signaling

The observation of linkage between receptors when applying unmatched targeted antagonists and agonists prompted us to better define potential antagonism, additivity, or synergies by combining different growth factors with pan-HER inhibitors and c-Met inhibitors. Because extensive analysis by median effects methods requires a much larger number of cells not readily available with zero passage primary tumor cells from patients, the HCC1954 cell line was chosen for this study due to its abnormal c-Met signaling in addition to abnormal EGFR (HER1) signaling determined by the CELsignia test. The HCC1954 cell line overexpresses HER2, but does not have abnormal levels of CELsignia HER2 score or demonstrate HER2 targeting drug sensitivity associated with abnormal levels of HER2 specific signaling [27] as shown in Additional File 1: Figure S3A-3C.

The CELsignia test was performed to obtain median effects data using HCC1954 cells by the Chou and Talalay method [26]. Since cells experience simultaneous signaling activations via different receptors under physiologic conditions, we first characterized combinations of different growth factors using the CELsignia test described above. The median effects analysis of growth factors showed antagonism (Combination Index, Ci, values > 1) for EGF when this growth factor was paired with HGF (Table 3; Additional File 1: Fig. S4A-4B and Fig.S5A. Similar results for growth factor antagonism between EGF and NRG, HER family, and c-Met receptors in primary cell samples were observed (Additional File 1: Table S3).

**Table3:** HCC1954 combination index data for HGF + EGF

HGF (pM)	EGF (pM)	Fraction activated (%)	Combination Index
50	300	92.8	1.43
10	60	58.9	1.81
2	12	25.7	1.25
0.4	2.4	8.7	0.8

We next used median effects data to analyze combinations of drugs on the growth factor cocktail. The combination index data (Table 4, Additional File 1: Fig. S5B) demonstrated that for this model cell line there is strong synergistic effect of combining the two drugs (Ci < 1) such that at 30 nM tepotinib and 60 nM neratinib, greater than 60% of the total HER/c-Met dysfunctional signal could be inhibited where the single drugs at these concentrations were not as efficacious. Where sufficient primary cell sample materials were available for more extensive median effects analyses, drug combination synergies against combined agonists were found (Additional File 1: Fig. S5C and Table S3).

**Table 4:** HCC1954 combination index data for neratinib and tepotinib on HGF and EGF

Tepotinib (nM)	Neratinib (nM)	Fraction inhibited (%)	Combination index
5	5	32	0.377 (n = 2)
10	10	41	0.37 (n = 2)
20	20	55	0.264 (n = 2)
30	60	62	0.062 (n = 2)

The data from HCC1954 cells was consistent with the observation in the primary breast cancer cells and therefore provides a correlative model for analysis of combination therapy in an in vivo animal model described below.

### Combinations of different pan-HER and c-Met inhibitors effectively block the HER-family and the c-Met receptor coactivated functions

Having established the signaling function test for c-Met, in addition to HER2, and the interactions between the growth factors and pan-HER and c-Met targeted drugs, we next applied the CELsignia test to determine any efficiency of combination treatment for the HER and c-Met receptors in patients with co-activated pathways. As opposed to in vitro, one variable at a time experimentation, cells in tissue encounter and respond to multiple perturbants in real time. To simulate this multiplexed perturbant condition and test the ability of the different targeted drugs to attenuate abnormal signaling from multiplexed agonists, patient breast cancer cells were stimulated with a growth factor cocktail of EGF, NRG1b, and HGF (N/E/H) to simultaneously initiate signaling via the HER-family and c-Met receptors. Neratinib (pan-HER inhibitor; 500 nM), tepotinib (c-Met inhibitor; 50 nM), or a combination of the two receptor antagonists were added to the cells prior to combined growth factor addition. Cell index signaling units were calculated relative to the baseline values obtained from cells that were not treated with either agonists or antagonists.

Data obtained from a representative patient cell sample shows that the combination treatment using the pan-HER and c-Met inhibitors was most efficient in blocking the simultaneous stimulation of pathways by the growth factor cocktail compared to the treatment with individual inhibitors (Fig. 5). Tables 5 and 6). As shown in Fig. 5, the patient cellular response in order of increasing efficacy to the inhibitory drugs was Tepotinib < Neratinib < Tepotinib + Neratinib. Further investigation for comparison of different inhibitor combination treatments was carried out with this patient cells and the percent inhibition for each combination is presented in Tables 5–6 where greater than 90% signal inhibition was demonstrated for all c-Met and pan-HER targeted combinations except lapatinib.

**Table 5:** Percent inhibition of the combined NRG/EGF/HGF cocktail signal with c-Met inhibitors against each listed pan-HER inhibitor on patient sample R66

Pan-HER inhibitors	Average inhibition (%) w/ different c-Met inhibitors
Poziotinib	100 (n = 3)
Neratinib	100 (n = 6)
Ibrutinib	99 (n = 6)
Dacomitinib	100 (n = 6)
Sapitinib	98 (n = 6)
Lapatinib	80 (n = 6)

**Table 6:** Percent inhibition of combined NRG/EGF/HGF cocktail signal with pan-HER inhibitors against each listed c-Met inhibitor on patient sample R66

c-Met inhibitors	Average inhibition (%) w/different HER inhibitors
Capmatinib	94 (n = 6)
Savolitinib	98 (n = 6)
Tepotinib	96 (n = 6)
Cabozantinib	99 (n = 6)
Crizotinib	100 (n = 6)

During the combined drug testing, we investigated the benefit of targeting HER1 specifically in combination with c-Met. The results showed that tepotinib (c-Met inhibitor), erlotinib (HER1 inhibitor), and neratinib (pan-HER inhibitor) have limited effectiveness as single drugs when HER and c-Met dysfunctional signaling pathways are co-activated (Table 7, Additional File 1: Fig. S6). The combination of HER1 and c-Met inhibitors caused about 50% reduction in signaling where maximum inhibition efficacy was observed in the presence of pan-HER and c-Met inhibitors that caused greater than 80% signal inhibition in the presence of the agonist cocktail (Table 7).

**Table 7:** CELsignia analysis of HCC1954 cells using indicated antagonists against a cocktail of agonists (NGR, EGF, HGF)

Drugs	HER/c-met inhibition (%)
Erlotinib (HER1i)	9.2 ± 10.3 (n = 2)
Tepotinib (c-METi)	− 8.8 ± 12.8 (n = 5)
Neratinib (pan-HERi)	14.6 ± 29.6 (n = 4)
Erlotinib + Tepotinib	47.6 ± 0.1 (n = 2)
Neratinib + Tepotinib	82.5 ± 20.1 (n = 4)

Errors listed are in standard deviation

### CELsignia test is a more sensitive and rapid test for detecting drug efficacy compared to the biological correlates

**Fig. 6:**
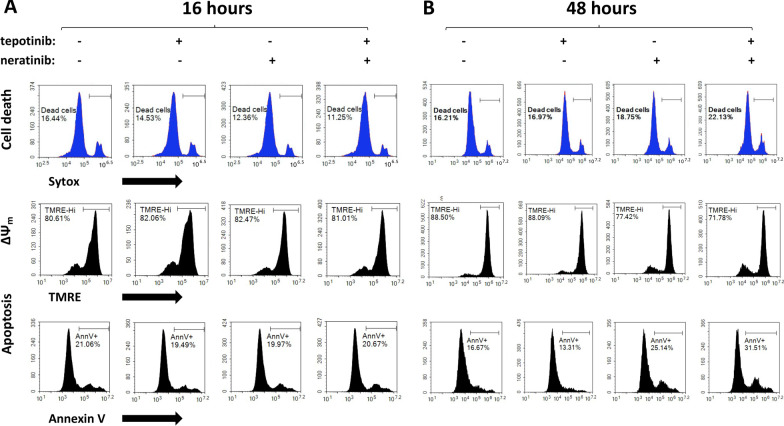
CELsignia test is a more sensitive and rapid test for detecting drug efficacy compared to the biological correlates. HCC1954 cells were seeded in collagen-fibronectin (CF) coated culture plates and treated 6 h later with tepotinib (250 nM) and neratinib (250 nM), either singly or in combination, as indicated. Cells were harvested and then analyzed by flow cytometry for the markers shown following a period of 16 h (**a**) or 48 h (**b**) of drug treatment. The data indicate relative insensitivity of flow cytometry markers to deteriorating cell health until at least 48 h after drug application. At 48 h, annexin v expression has increased by nearly twofold for cells treated with combined pan-HER and cMET targeted drugs

We next examined the correlation of our CELsignia based real-time live cell signaling responses with commonly used research markers of drug-induced cell stress using single-cell flow cytometry analysis. Assays that require higher drug levels for longer periods of time run the risk of off-target and less specifically determinative effects. For these experiments, HCC1954 (HER2 + , c-Met inhibitor-sensitive) [28] cell results are presented here. Initial experiments in HCC1954 indicated that following 16-h drug treatment (i.e. the time frame in which drug sensitivity can be identified by CELsignia), both tepotinib and neratinib failed to induce appreciable changes in levels of cell death or other physiologic health markers as compared to untreated controls, when used either singly or in combination at different doses at timepoints less than 16 h (Fig. 6a, Additional File 1: Fig S17). Moreover, drug treatments also failed to trigger significant changes in the apoptotic marker phosphatidyl-serine (Annexin V) or mitochondrial membrane potential (TMRE, Fig. 6A) in less than 16 h. Similar results were also obtained in negative control cells, BT-20 and MDA-MB-231(Additional File 1: Figs.S7-S12), having normal HER or cMet-linked signaling in the impedance test platform (Additional File 1: Fig. S13). The normal CELsignia signaling results for these cell lines are in agreement with extensive previous reports whereby the cells’ increase in EGFR and c-Met phosphorylations upon EGF and HGF additions demonstrated by western blotting do not affect cell viability and proliferation [29, 30]. Similar to HCC1954, a 16-h drug treatment period in these cell lines with neratinib and/or tepotinib failed to reveal detectable effects on cell death, apoptosis, or mitochondrial membrane potential.

Next, we extended the sampling time for flow cytometry marker experiments for prolonged treatment of HCC1954 cells with tepotinib and neratinib (48 h using 250 nM each drug vs. CELsignia at 50 nM each drug, either singly or in combination) to determine if significant marker changes occurred for the HCC1954 sensitivity to these drugs that is detected sooner by CELsignia in < 24 h. Under the extended time point conditions, enhanced cell death, decreased mitochondrial potential and increased annexin V positivity were detected at least 48 h after drug addition in the presence of both neratinib and more significantly for the neratinib/tepotinib cocktail, but not following tepotinib treatment alone (Fig. 6b). This result corroborates the CELsignia test result and verifies that the cells enter programmed cell death when their oncogenic hypersignaling is attenuated with the matched targeted therapies. However, despite an increased concentration and time of drug treatment (up to 72 h), bio-correlative markers of cytotoxicity were not significant in other cell lines (BT474, A549) that were clearly identified by CELsignia to be normal signaling via cMet and HER family (Additional File 1: Fig. S14-S17). Collectively, the results demonstrate that drug sensitivity of cancer cells identified by CELsignia precedes, and in some cases is challenging to detect after greater than 48 h of treatment, using widely employed cellular ‘point of no return’ canonical bio-correlates (e.g. loss of mitochondrial membrane integrity by TMRE, apoptosis by Annexin V, and loss of plasma membrane integrity by Sytox).

### Combination of pan-HER and c-Met inhibitors impact intracellular markers by flow cytometry of cell health and adhesion

**Fig. 7:**
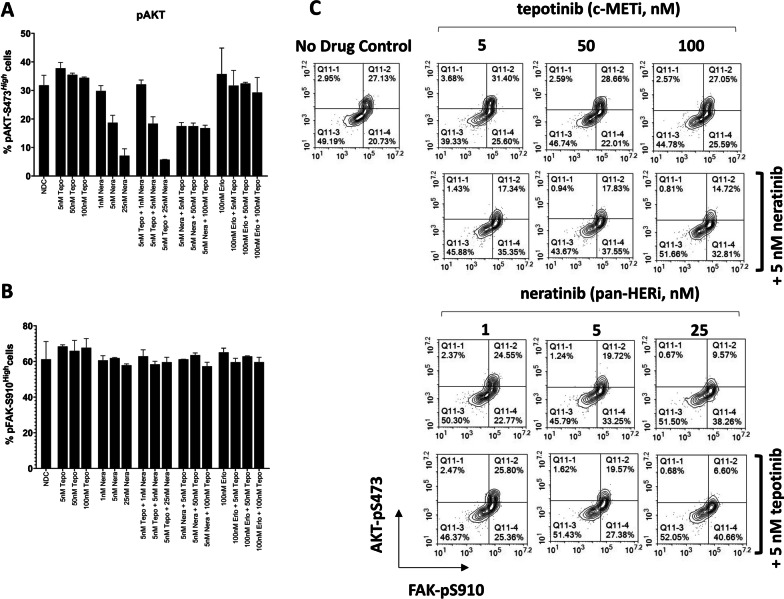
Single-cell analysis of AKT and FAK pathway activation does not reveal the HER-dependent differential sensitivity or drug synergy of HCC1954 cells to pan-HER and c-MET inhibitor combinations identified by CELsignia. HCC1954 cells were treated with c-MET (tepotinib) and pan-HER (neratinib) inhibitors, either singly or in combination, at the doses indicated for 16 h as described in Fig. 6 and then analyzed by intracellular phospho-flow cytometry. **a**, **b** The mean and standard deviation of levels of pAKT and pFAK in gated live cells following drug treatment from two experiments is shown. **c** Cells were treated with increasing doses of tepotinib or neratinib, administered singly or in combination as indicated, and analyzed for pAKT and pFAK expression patterns within single cells. At least 10,000 events were acquired per sample and data shown is representative of two experiments

In order to look for biocorrelative insight underlying CELsignia impedance-based dysfunctional signaling phenotypes for at least one specific cell sample, we analyzed the phosphorylation status of two canonical intracellular signaling markers related to cell signaling and survival (AKT-pS473) and one example of adhesion complex-regulated adhesion/migration (FAK-pS910) [31, 32] in HCC1954 cells treated for 16 h with tepotinib and/or neratinib. The data presented in Fig. 7 demonstrate that a dose-dependent decline in the frequency of pAKT^high^ cells occurs with neratinib, when used singly and in combination with tepotinib (Fig. 7a). In contrast to pAKT, the distribution of pFAK^high^ cells is not significantly altered by neratinib (or by neratinib/tepotinib combination treatments) (Fig. 7b). When considering the two-dimensional flow cytometry plots at a single-cell type level, we unexpectedly found neratinib treatment resulted in a dose-dependent shift in the hierarchy of HCC1954 AKT/FAK signaling phenotypes (Fig. 7c). Specifically, HER inhibition, alone or in combination with tepotinib, substantially decreased the proportion of pAKT^high^pFAK^high^ cells and increased the proportion of the pFAK^high^pAKT^low^ sub-population (Fig. 7c and Additional File 1: Fig. S16). When the pFAK^high^pAKT^low^ change is found co-expressed in a significant population of cells, the interpretation is that the cells are undergoing major deleterious effects leading to apoptosis (27).

In contrast, tepotinib, when applied as a single agent, exerted no detectable dose-dependent effect on pFAK and pAKT population distributions. At higher doses of tepotinib, in the presence of a synergistic dose (5 nM) of neratinib (Fig. 7b and Additional File 1: Fig. S16) change in pAKT became significant though pFAK did not change (i.e. pFAK^high^pAKT^low^ population distribution increased). This change to pAKT is likely entirely due to 5 nM neratinib and this flow cytometry analysis of these two markers is not demonstrating any synergy of combining the two drugs as was easily demonstrated and quantified with the CELsignia test. These findings indicate that the pattern of HER-dependent differential sensitivity of HCC1954 cells to c-Met inhibitors identified by CELsignia correlates with unique effects of neratinib and combinations with tepotinib on both the AKT and FAK signaling pathways for this particular cell line.

### Combination of pan-HER and c-Met inhibitors effectively reduce tumor size in the xenograft model

**Fig. 8:**
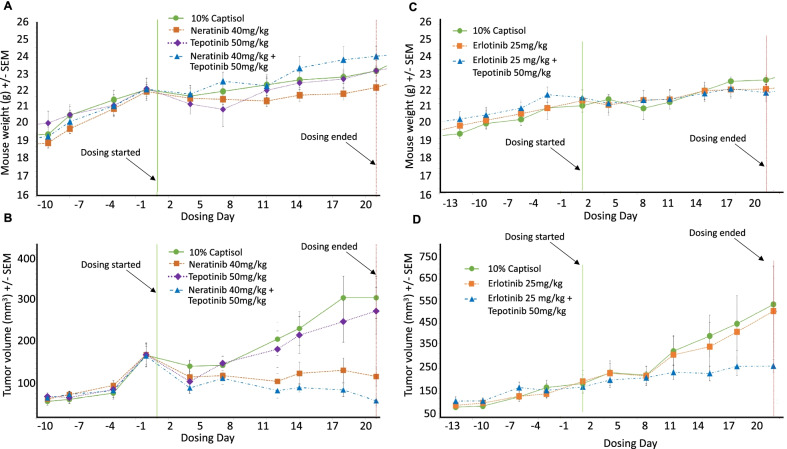
Combination of pan-HER and c-Met inhibitors effectively reduce tumor size in the xenograft model. Activity of c-MET and HER specific antagonists in a HCC1954 a NSG mouse xenograft model. Ten mice cohorts were dosed orally QD for 21 days. Mouse weights (Panels A and C) and tumor size (Panels B and D) were recorded every 3 days

To verify the results obtained from our in vitro analysis using both primary cells and the HCC1954 cell line, we next carried out an in vivo experiment using the HCC1954-xenograft model. Female NSG mice, 4–5 weeks old, were injected subcutaneously in the left mammary fat pad with two million HCC1954 cells. After the average tumor volume reached 150 mm^3^ the mice were randomly assigned into six cohorts of 10 mice each (n = 10). Each of the experimental mice were dosed by oral gavage with 100 µL dosing solution daily (QD) for 21 days (21 doses) [33–35]. The six cohorts included the vehicle control group dosed with10% Captisol and five treatment groups including neratinib, tepotinib, erlotinib, erlotinib + tepotinib, or neratinib + tepotinib (Table 8) To ensure that the animals maintained consistent weight gain as compared to the Captisol-treated arm (control), the body weight was measured throughout the course of the experiment (Fig. 8a). Mice that suffered loss of body weight were not included in data capture and analysis (Table 8). In order to determine the effects of the pan-HER and c-Met antagonists on tumor size, the tumor volume (in mm^3^) was measured and plotted as shown in Fig. 8b. The data revealed a maximal effect on tumor reduction when mice were treated with a combination of HER and c-Met receptor antagonists when compared with the mice treated with the drugs individually (Table 9). We observed a 71% reduction in tumor size in mice treated with pan-HER + c-Met antagonist combination compared to 51% reduction when treated with HER1 + c-Met antagonist combination. The data obtained from the in vivo experiment correlated with the CELsignia test result and further established the importance of treating tumors with a combination of antagonists targeting the pan-HER- and c-Met signaling pathways. When the drug neratinib was administered as a single agent in the xenograft study, the data showed significant tumor reduction (54% vs control, Table 9) compared to the in vitro impedance signaling test (14.6% Table 7) when neratinib was tested upon a combination of three growth factors. Differences in concentrations of the growth factors in the in vitro test and the biological differences in the abilities of the murine proteins to activate human receptors [36] in the cell line are likely significant factors in the differences for these outcomes.

**Table 8:** HCC1954 Xenograft model: experimental design

Experiment	Cohort	n	Drug	Dose (mg/kg)	Dosing frequency	Number of doses
A	1	10	Vehicle	0	QD	21
	2	10	Neratinib	40	QD	21
	3	10	Tepotinib	50	QD	21^#^
	4	9*	Neratinib + Tepotinib	40 + 50	QD	21
B	1	7^^^	Vehicle	0	QD	21
	2	8	Erlotinib	25	QD	21
	3	8	Erlotinib + Tepotinib	25 + 50	QD	21

*1 mouse experienced weight loss and diarrhea and was found dead on treatment day 13

^#^Due to weight loss, 1 mouse given 3 day dose holiday due, and 1 mouse given 7 day dose holiday

^^^1 mouse euthanized on treatment day 8 due to severe weight loss

**Table 9:** Percent tumor reduction in HCC1954 xenograft model treated with HER-specific and c-MET specific antagonists

Drug arm vs. comparator arm	Tumor reduction (*t* test)
Tepotinib versus control	10% (*p* = 0.780)
Neratinib versus control	54% (*p* = 0.003)
Neratinib + tepotinib versus control	71% (*p* = 0.0003)
Neratinib + tepotinib versus neratinib	37% (*p* = 0.05)
Erlotinib versus control	5% (*p* = 0.870)
Erlotinib + tepotinib versus control	51% (*p* = 0.110)

## Discussion

The CELsignia test is focused on reporting a clear and clinically actionable result—the abnormal coordinated dysfunctional signaling problem in cancer patients and the potential for successful combined targeted therapy intervention. The test can assess coincidental agonisms and antagonisms from different simultaneous sources.

One remarkable distinction is that the test encompasses the effects of important factors that are not considered in other clinical tests including signaling time, local intra-cellular organization of proteins and their specific activities and contributions to signaling complexity [37, 38]. We highlight that the clinically actionable test result is not dependent on reporting what the root molecular causes are, of which there may be many, nor deciphering how a physician is to interpret the test result.

Apparently, good molecular tools for clinical application do not yet exist to quantify the detailed inner workings (time, specific activity, location) of all the proteins in tumor cells at the level required to predict how to treat the patient. We developed the live cell CELsignia clinical test for quantifying dysfunctional signaling and then applied basic scientific process to pre-clinically verify and identify an estimated cutoff of signaling amount and the prevalence in a statistically significant population of 79 histopathologically HER2-negative breast cancer patients with concomitant c-Met and HER family-driven signaling activity.

Many studies have sought to explain and treat the co-involvement of ErbB family and c-Met receptors in driving cancer [39]. The problem of determining targeted treatment for an active single receptor dysfunction is exacerbated when the receptor cooperates with other proteins in unexpected ways to reduce or eliminate any benefit of the first single agent such as has been described (Fig. 5) with this study for HER family and c-Met.

Overactive HER2 and HER2 heterodimerization with many other receptor types is a well-known problem in cancer and there are many drugs that can be clinically effective when the signaling dysfunction is actually present and occurring at relevant levels to drive the disease. Determining in which patients this is happening and determining the most efficacious points to inhibit the abnormal signaling with targeted therapies has been elusive. This is highlighted by reports that show in some cases effective targeted therapies such as pertuzumab can act as an artificial ligand to promote activation of ERK signaling [40]. Unintended activation of linked receptors upon treatment with a drug is found in Fig. 5 of this study using the CELsignia test.

Watson and Gray demonstrated the importance of independent HER and c-Met involvement in individual drug resistance for some patients potentially due to the presence of agonists in the tissue microenvironment [3].

Engelman et al. linked increased c-Met signaling to resistance to gefitinib via activated ERBB3 [6] where c-Met amplification leads to ERBB3 phosphorylation and PI3K activation in an EGFR- and ERBB2-independent manner. Their studies suggested that ERBB3-mediated activation of PI3K/Akt might be a common feature of cancer cells that have c-Met amplification.

In vitro analyses such as those described above, have prompted several clinical trials to test combinations of targeted therapeutics to treat ErbB co-involved c-Met cancer to little benefit. Several studies in the literature demonstrate the challenges with using RNA or protein levels as predictive markers for clinical utility in individual patient drug assignment [41, 42].

The adaptive potential of signaling complexity is one of the most likely explanations why previous trials to identify subpopulations of responding patients before treatment with HER/c-Met associated disease have not led to significant positive outcomes. Various RTK and, in particular the ErbB family and the c-Met receptor, comprise a nexus of two important cellular function control pathways, MAPK and PI3K. These pathways have multiple layers of control circuitry that enable cells to adapt to various microenvironment or chemical perturbations and still continue to supply energetic and anabolic function. The control mechanisms rely on specific activities of different forms of these proteins and other complexities such as kinetics of differential phosphorylation, and regio-spatial and temporal contexts within an individual patient’s tumor cells [43]. For example, several reports link c-Met activation mechanism and differential specific phosphorylations to receptor stabilization at the cell membrane surface, internalization to different locations within the cell, and or degradation of the receptor thus affecting the c-Met receptor signaling as well as the signaling function of other linked receptors or those receptors in close proximity [37, 38, 44, 45].

Studies have reported on the linkage of cell signaling to morphological changes and changes at the cell surface involving different adhesion complexes that are the strongest contributors to impedance changes that can be detected by a biosensor that is applied for the CELsignia test results reported in this study [46–48]. Using cell impedance testing,

we confirmed the specificity of the agonist response by applying c-Met inhibitors (tepotinib, capmatinib, crizotinib, savolitinib, and cabozantinib), a HER1 inhibitor (erlotinib), pan-HER inhibitors (neratinib, ibrutinib, dacomitinib, sapitinib, lapatinib, and poziotinib), and combinations of these therapies using the CELsignia test on patient live tumor cell samples. IC_50_ values of each of the six pan-HER inhibitors and five c-Met inhibitors are reported indicating many efficacious inhibitors when applied to cell samples actually having combined signaling dysfunction.

We characterized the extent of signaling complexity and cross-talk between simultaneous treatments with multiple HER family and c-Met agonists and antagonists in live tumor cell samples obtained from three different primary breast cancer patient tumor specimens and show very strong synergy between the simultaneous combined pan-HER and c-Met inhibition. These results suggest that disruption of one receptor type signaling must leave some fraction of the other type of receptor available for significant dysfunctional signaling.

In this study, to further elucidate the role of c-Met signaling and its co-involvement with HER family signaling as a cancer driver, we demonstrated how growth factor receptor signaling is surprisingly strongly antagonistic between the HER family and c-Met when studying the combined stimulations. Our data show the strong synergy of the combination of pan-HER inhibition and c-Met inhibition against simultaneous abnormal receptor signaling between these receptors. Furthermore, the Chou and Talalay analyses using cell impedance testing demonstrate that the combination of growth factor antagonisms were mutually exclusive as was recently published in another report [45]. Thus, when one of these growth factors is activated, a cell mechanism (e.g. endocytosis) prevents further test signal development via the other receptor type. However, this reduction in further agonism does not prevent signaling by a linked receptor type and this signaling can be disrupted with strong synergy by combined targeted drug additions.

Finally, we provide evidence that targeted drugs disrupting the abnormal levels of HER/cMET signaling in tumor cells detected quantitatively in 4 h by CELsignia leads to mouse xenograft tumor reduction and other orthogonal biomarkers indicative of declining function and programmed cell death (Fig. 6) at 48 h after drug addition.

## Conclusions

The findings of this report support identification of a significant sub-group of breast cancer patients with cooperating pan-HER and c-Met signaling dysfunction that may respond to treatment with a combination of pan-HER and c-Met inhibitors. Current tools provide a static assessment of pathway components at specific timepoints, but they have not provided a dynamic analysis quantifying dysfunctional signal transduction or accurately identifying sensitive disruption nodes for individual patients. The CELsignia, functional live tumor cell test described herein provides a dynamic assessment of cooperative signaling pathway activity. Using this test, a sub-set of HER2 negative breast cancer patients with cooperative and dysregulated HER family and c-Met pathways was identified. Two clinical trials to evaluate treatment response of this breast cancer patient sub-set to these combined pathway inhibitors identified by the CELsignia test are underway.

## Supplementary Information

**Additional file 1**. Patient characteristics based on age, stage of cancer, tumor histology, and expression of estrogen receptor in tumor cells. All patients enrolled in this study expressed normal levels of HER2 receptor.

## Data Availability

All data are available in the main text or the supplementary materials. The dataset analyzed during the current study are available from the corresponding author upon reasonable request.

## Abbreviations

CELsignia: CELsignia multi-pathway signaling function
c-Met: Hepatocyte growth factor receptor
HER: Human epidermal growth factor receptor
EGFR: Epidermal growth factor receptor
MAPK: Mitogen activated protein kinase
PI3K: Phosphoinositide-3-kinase
PFS: Progression free survival
IHC: Immunohistochemical analysis
FISH: Fluorescence in situ hybridization
AAHRPP: Accreditation of Human Research Protection Programs
NRG1b: Neuregulin 1b
NRG: Neuregulin
EGF: Epidermal growth factor
HGF: Hepatocyte growth factor
N/E/H: NRG1b + EGF + HGF
ROC: Receiver operator characteristic
