# Supplementary material for: Functional signaling test identifies HER2 negative breast cancer patients who may benefit from c-Met and pan-HER combination therapy
Source: Cell Commun Signal. 2022 Jan 8;20:4. doi: 10.1186/s12964-021-00798-9 (PMC8742957; doi:10.1186/s12964-021-00798-9)
Supplement: Supplementary file 2 — Additional file 1. Patient characteristics based on age, stage of cancer, tumor histology, and expression of estrogen receptor in tumor cells. All patients enrolled in this study expressed normal levels of HER2 receptor. [file 12964_2021_798_MOESM2_ESM.pdf]

Ian A MacNeil, Salmaan A Khan, Adrish Sen, Sajjad M Soltani, David J Burns, Brian F Sullivan, and Lance G Laing*

Celcuity, Inc. 16305 36th Ave N, Suite 100, Minneapolis, MN 55446

***Corresponding Author**

Email: LLaing@Celcuity.com

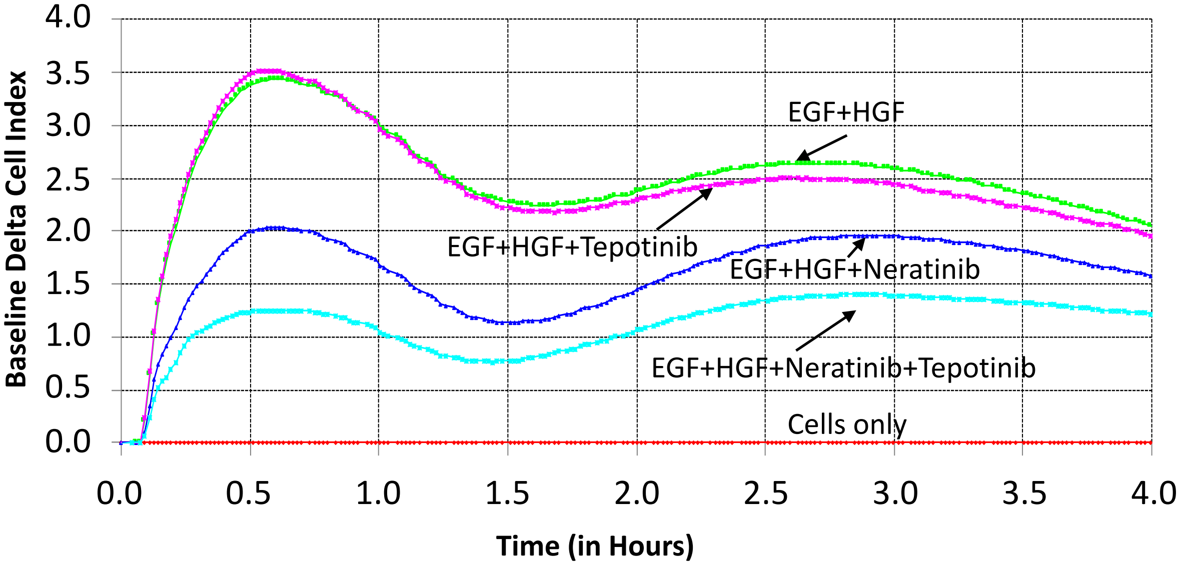

Fig. S1: An example of impedance time course data used in median effects analysis of synergy for the antagonists is presented for HCC1954 cells treated with either 20nM tepotinib (E+H+T), 20nM neratinib (E+H+N), or a combination of the two drugs each at 20nM (E+H+N+T), followed by the addition of a combination of HGF and EGF. The figure also includes a HCC1954 cell sample that has had no agonist or antagonist addition (Cells only) and a HCC1954 cell sample that has only the agonists, HGF and EGF, added in combination (E+H).

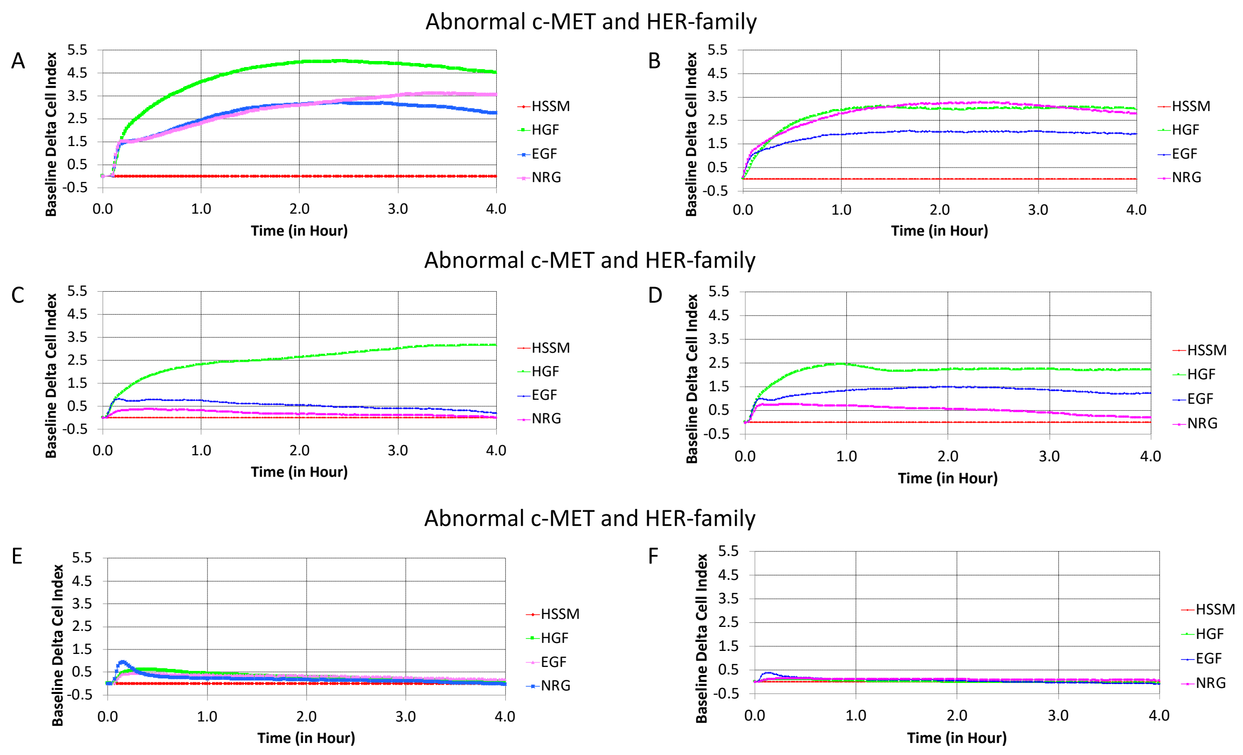

Fig. S2: Impedance time course data from 6 patient samples stimulated with NRG (pink line), EGF (blue line), HGF (green line) or no growth factors (HSSM, red line). Panels A&B) Abnormal c-Met and Abnormal HER-family receptor signaling. Panels C&D) Abnormal c-Met and normal HER2 receptor signaling. Panels E&F) normal c-Met and normal HER-family receptor signaling.

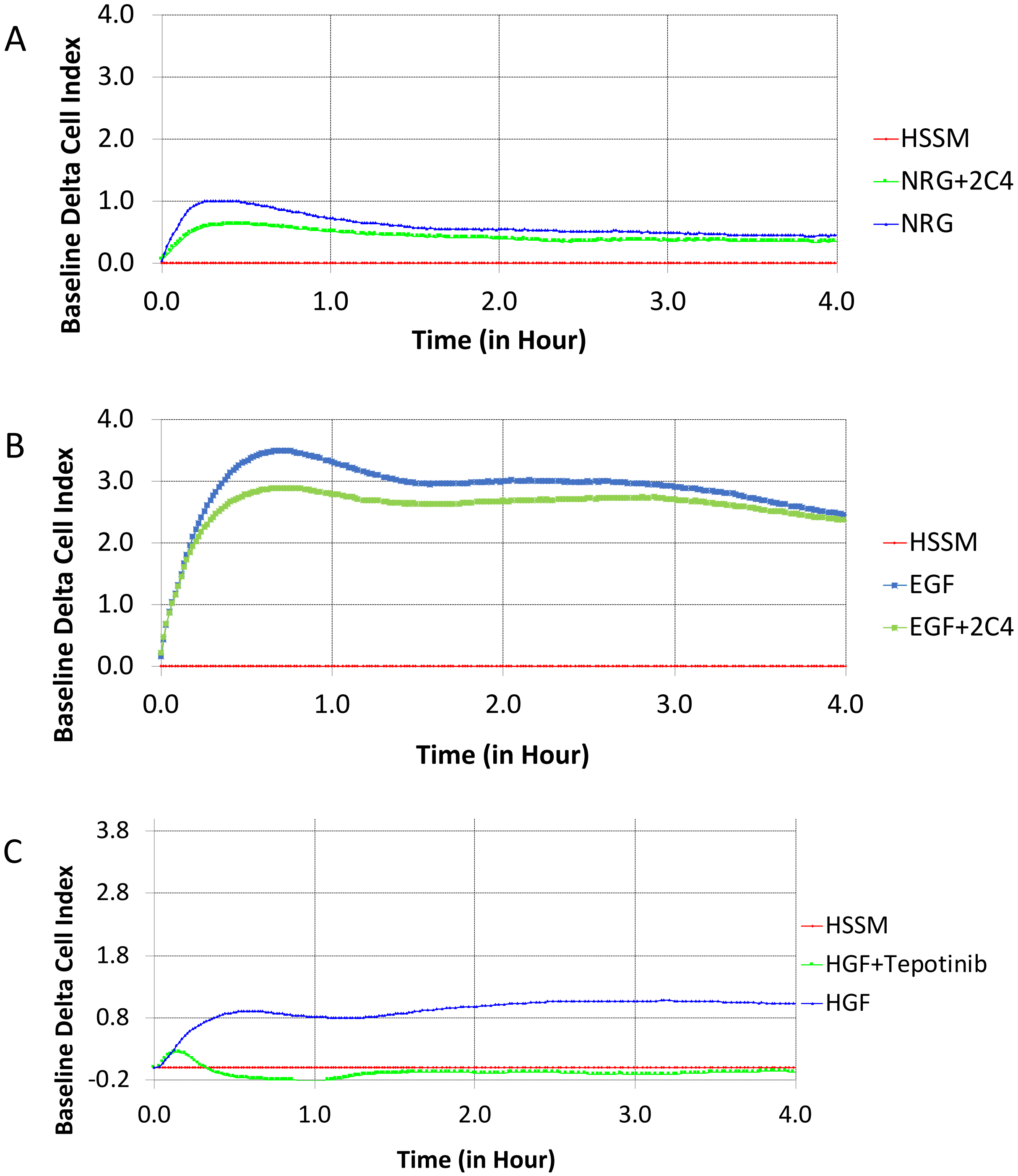

Fig. S3: The CELsignia test characterizes the level of HER2 and c-Met signaling in HCC1954, a HER2 overexpressing cell line. Impedance timecourse graphs are shown on

matching Y-axis scales in the three panels to highlight differences in signals. Analysis of the HER2 involvement in EGF and NRG signaling for HCC1954 demonstrates that HER2 stimulated signaling is not abnormally large compared to EGF and HGF stimulated signaling in this cell line. Using the same ordinate axis scale, three panels are presented for HCC1954 instrument data in response to addition of growth factors compared to no growth factor addition (HSSM, red line). A) CELsignia impedance timecourse of NRG stimulation with and without added HER2 receptor dimerization antibody. The difference between the two NRG treated wells represents the amount of HER2 involvement in NRG signaling. B) CELsignia impedance timecourse of EGF stimulation with and without added HER2 receptor dimerization antibody. The difference between the two EGF treated wells represents the amount of HER2 involvement in EGF signaling. C) CELsignia impedance timecourse of HGF stimulation with and without added c-Met targeting small molecule, tepotinib. The application of low concentrations of agonist (50pM) and matching targeted antagonist (50nM) verify the specificity of the test signal. The difference between the two NRG treated wells represents the amount of HER2 involvement in NRG signaling. An analysis of the HER2 involvement in EGF and NRG signaling for HCC1954 demonstrates that HER2 stimulated signaling is not abnormally large compared to EGF and HGF stimulated signaling

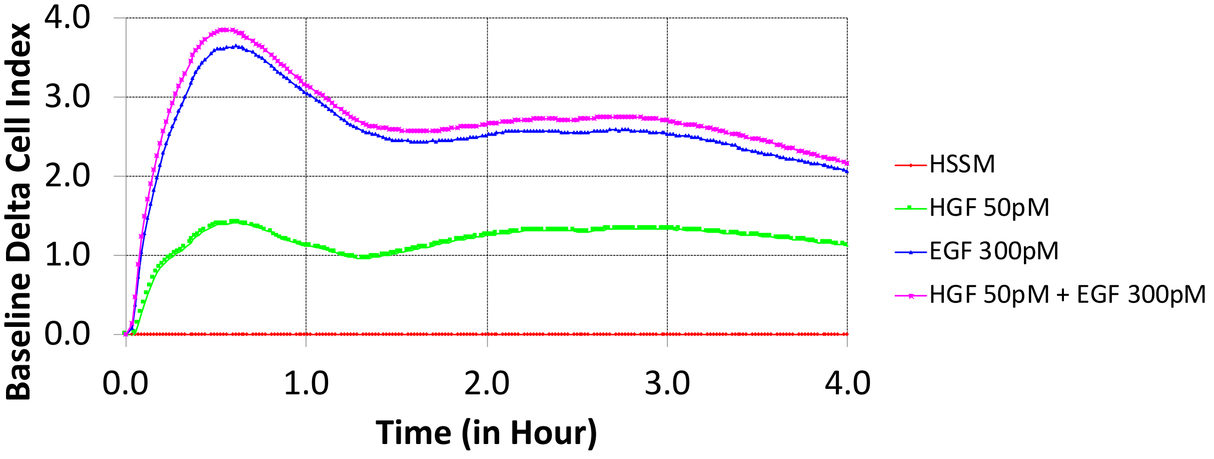

Fig. S4: An example of impedance time course data used in median effects analysis of interactions for the agonists is presented for HCC1954 cells treated with single growth factors (EGF, HGF) or combinations of EGF and HGF. The figure also includes a HCC1954 cell sample that has had no agonist or antagonist addition (HSSM, red line). The data are indicative of antagonism between the growth factors when multiple paired titration points are compiled.

A

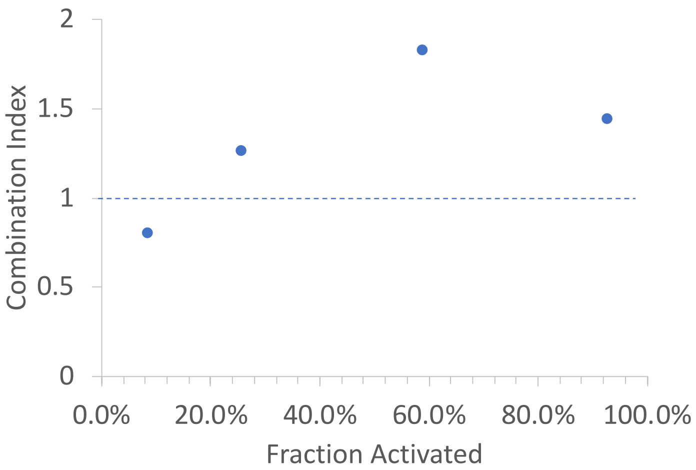

B

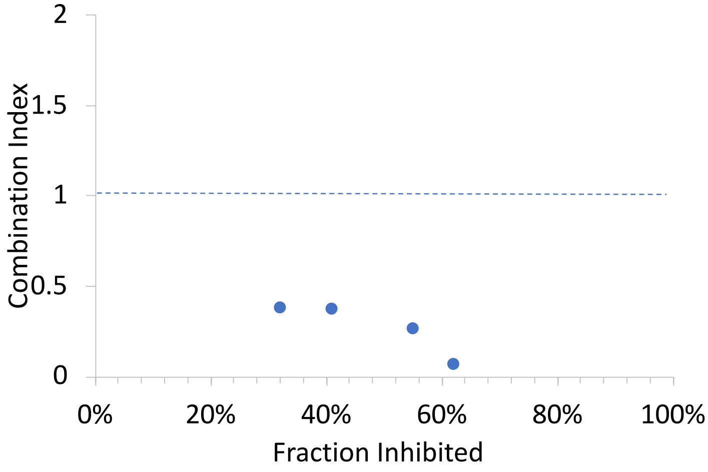

C

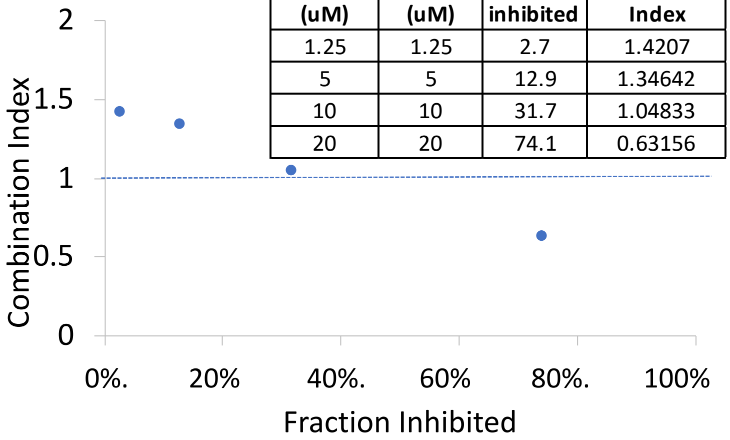

Tepotinib (uM)	Neratinib (uM)	Fraction inhibited	Combination Index
1.25	1.25	2.7	1.4207
5	5	12.9	1.34642
10	10	31.7	1.04833
20	20	74.1	0.63156

Fig. S5: Median effects plots. A) Agonist median effects plot of the combination of HGF and EGF on HCC1954. Percent activated fraction numbers are listed in Table 3. When CI>1, antagoniosm is indicated B) Antagonist median effects plot of HCC1954. The two inhibitors (neratinib and tepotinib) were paired at IC50 concentrations as determined from the impedance test analysis of the single growth factor (Tepotinib + HGF has tepotinib IC50=7 nM and neratinib + EGF has neratinib IC50=15 nM), and thus a 1:2 tepotinib to neratinib ratio was selected for the median effects treatments. Dose escalations of combined drugs were tested at the paired inhibitor ratios on combined EGF and HGF stimulations according to the method of Chou and Talalay. CI<1 is indicative of synergy. C) Antagonist median effects plot of patient sample C753. Percent inhibited fraction and Combination Index numbers are listed in the inset. CI= 1 is indicative of additivity; CI<1 is indicative of synergy.

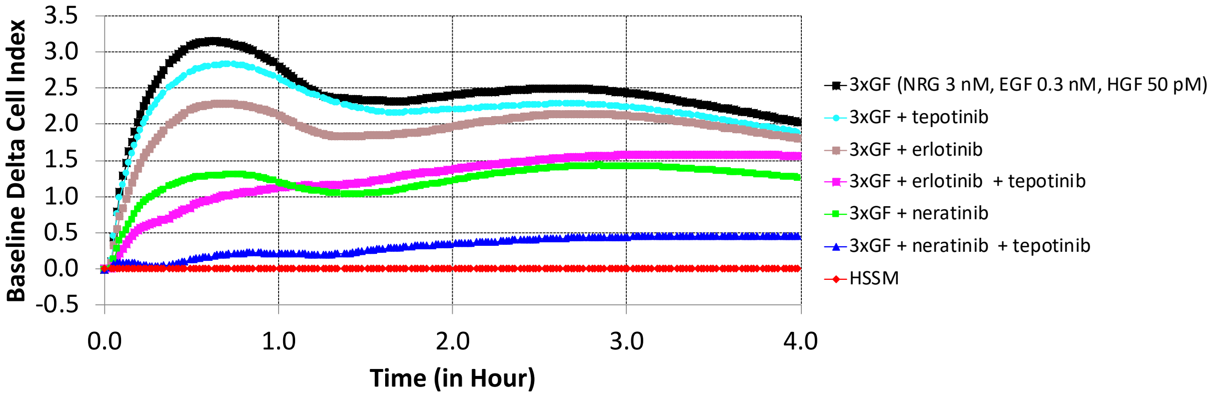

Fig. S6: Impedance time course example for antagonist effects on a cocktail of agonists (NRG, EGF and HGF) for HCC1954 used to derive data in Table 7 (N of 4 experiments). Also included on the plot timecourse are cells only (HSSM, red line) untreated by agonist or antagonist.

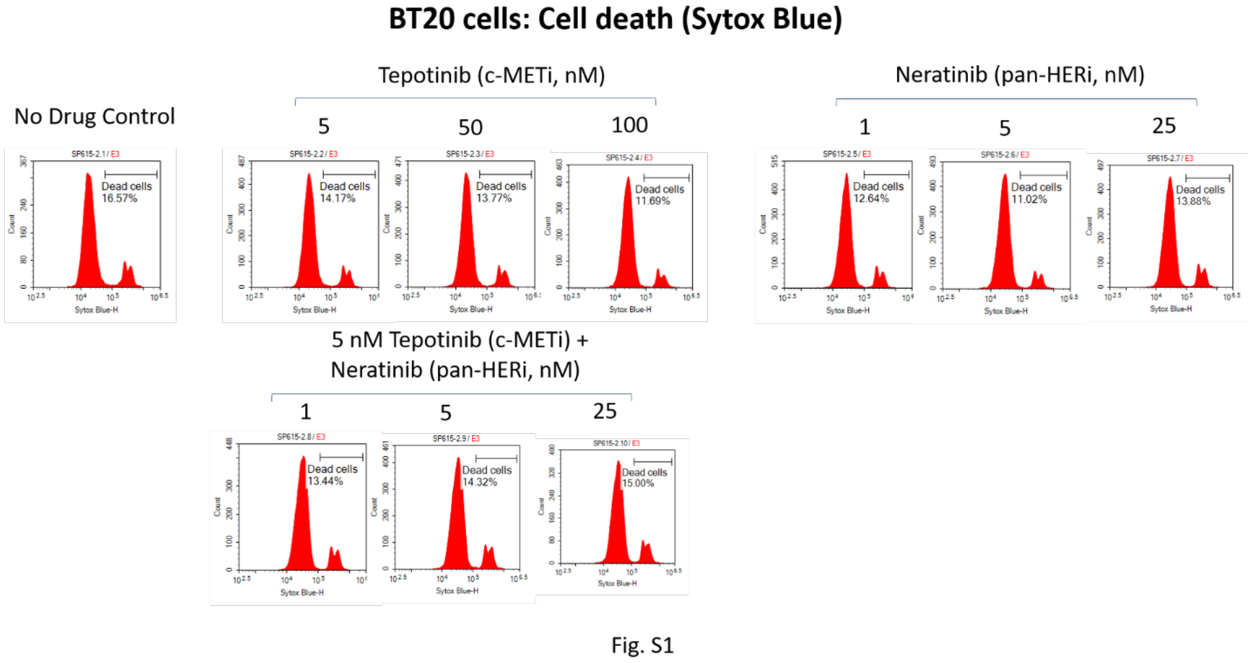

Figure S7. CELsignia test is more sensitive and rapid for detecting anti-cancer drug efficacy compared to biological correlates – Normal signaling cell line, BT-20. BT-20 cells were normal signaling for HER and c-Met by the CELsignia test. To test flow cytometry markers for correlation a test was setup following the dose and time parameters used for the CELsignia test. The cells were seeded in collagen-fibrinogen (CF) coated culture plates and treated 6h later with tepotinib (250 nM) and neratinib (250 nM), either singly or in combination, as indicated. Cells were harvested and then analyzed by flow cytometry for the markers shown following a period of 16h drug treatment. BT-20 cells do not respond in this timeframe to these drugs according to the Sytox blue marker.

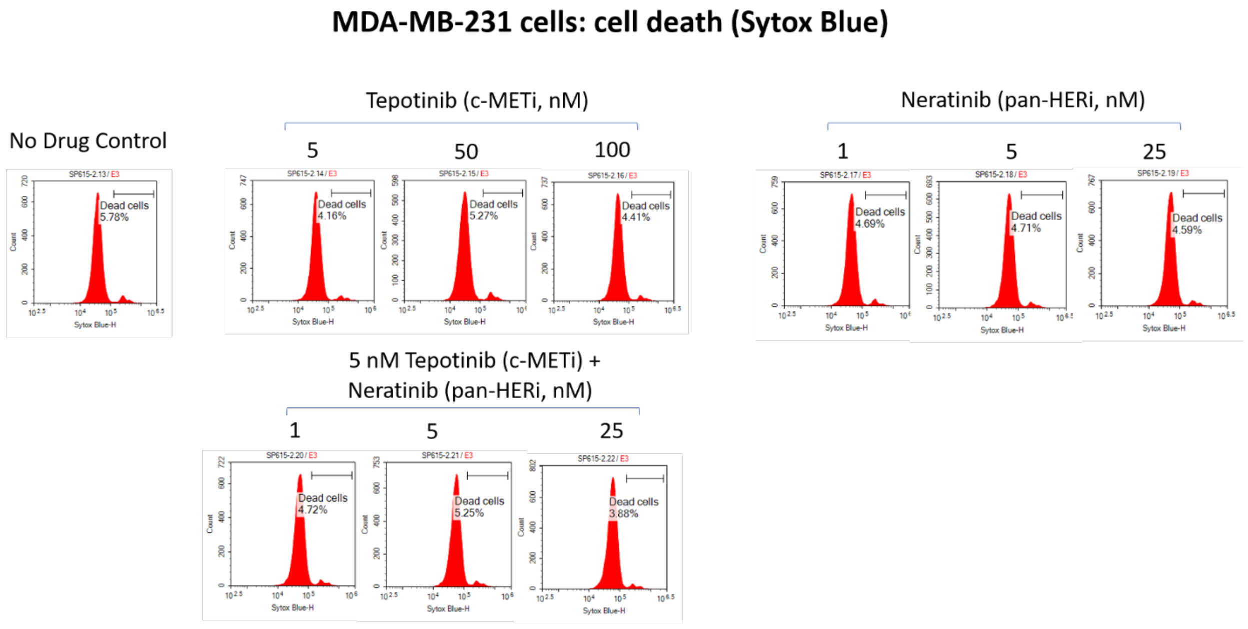

Fig. S2

Figure S8. CELsignia test is more sensitive and rapid for detecting anti-cancer drug efficacy compared to biological correlates – Normal signaling cell line, MDA-MB-231. MDA-MB-231 cells were normal signaling for HER and c-Met by the CELsignia test. To test flow cytometry markers for correlation a test was setup following the dose and time parameters used for the CELsignia test. The cells were seeded in collagen-fibrinogen (CF) coated culture plates and treated 6h later with tepotinib (250 nM) and neratinib (250 nM), either singly or in combination, as indicated. Cells were harvested and then analyzed by flow cytometry for the markers shown following a period of 16h drug treatment. MDA-MB-231 cells do not respond in this timeframe to these drugs according to the Sytox blue marker.

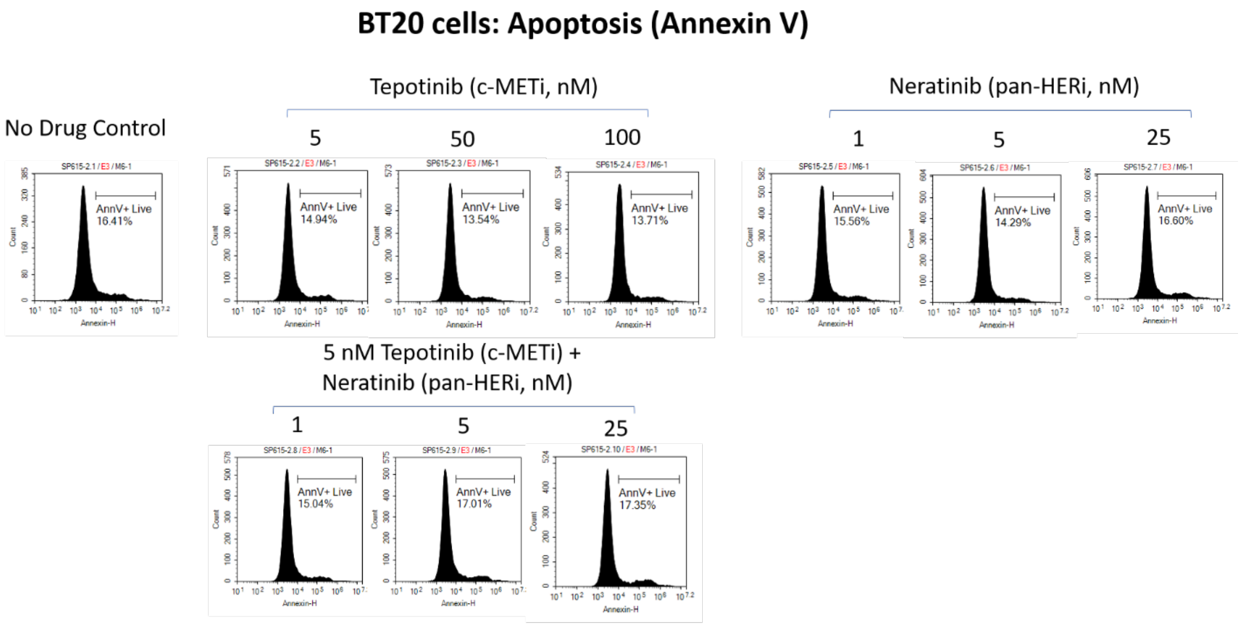

Fig. S3

Figure S9. CELsignia test is more sensitive and rapid for detecting anti-cancer drug efficacy compared to biological correlates – Normal signaling cell line, BT20 continued. BT-20 cells were seeded in collagen-fibrinogen (CF) coated culture plates and treated 6h later with tepotinib (250 nM) and neratinib (250 nM), either singly or in combination, as indicated. Cells were harvested and then analyzed by flow cytometry for the markers shown following a period of 16h drug treatment. BT-20 cells do not respond in this timeframe to these drugs according to annexin V marker.

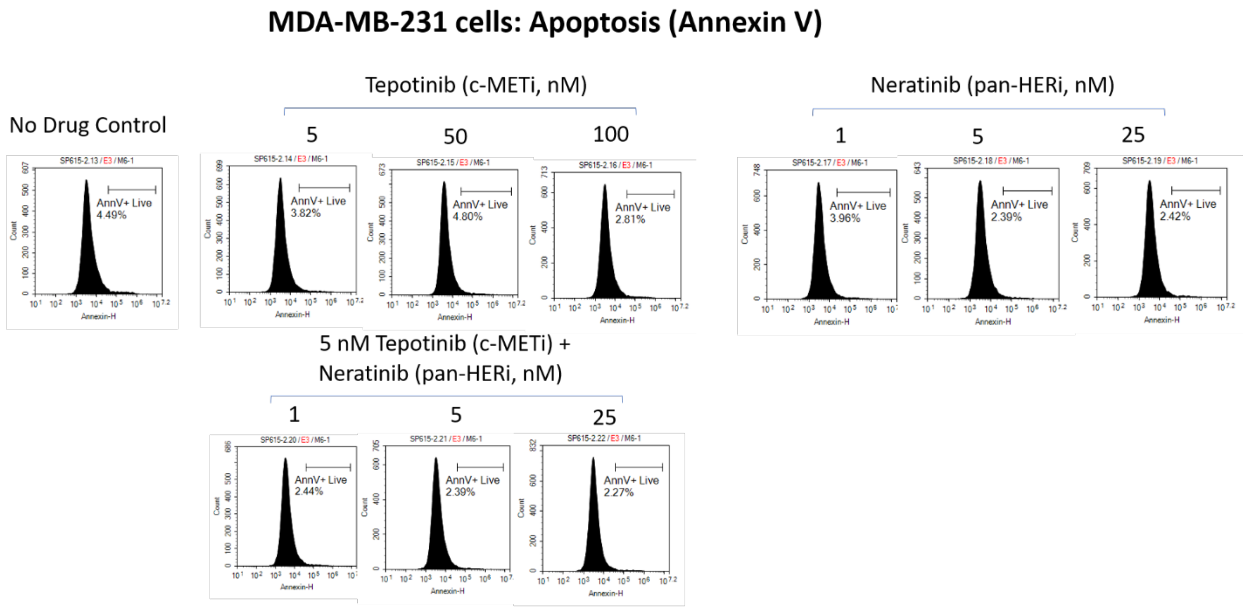

Fig. S4

Figure S10. CELsignia test is more sensitive and rapid for detecting anti-cancer drug efficacy compared to biological correlates – Normal signaling cell line, MDA-MB-231 continued. MDA-MB-231 cells were seeded in collagen-fibrinogen (CF) coated culture plates and treated 6h later with tepotinib (250 nM) and neratinib (250 nM), either singly or in combination, as indicated. Cells were harvested and then analyzed by flow cytometry for the markers shown following a period of 16h drug treatment. MDA-MB-231 cells do not respond in this timeframe to these drugs according to annexin V marker.

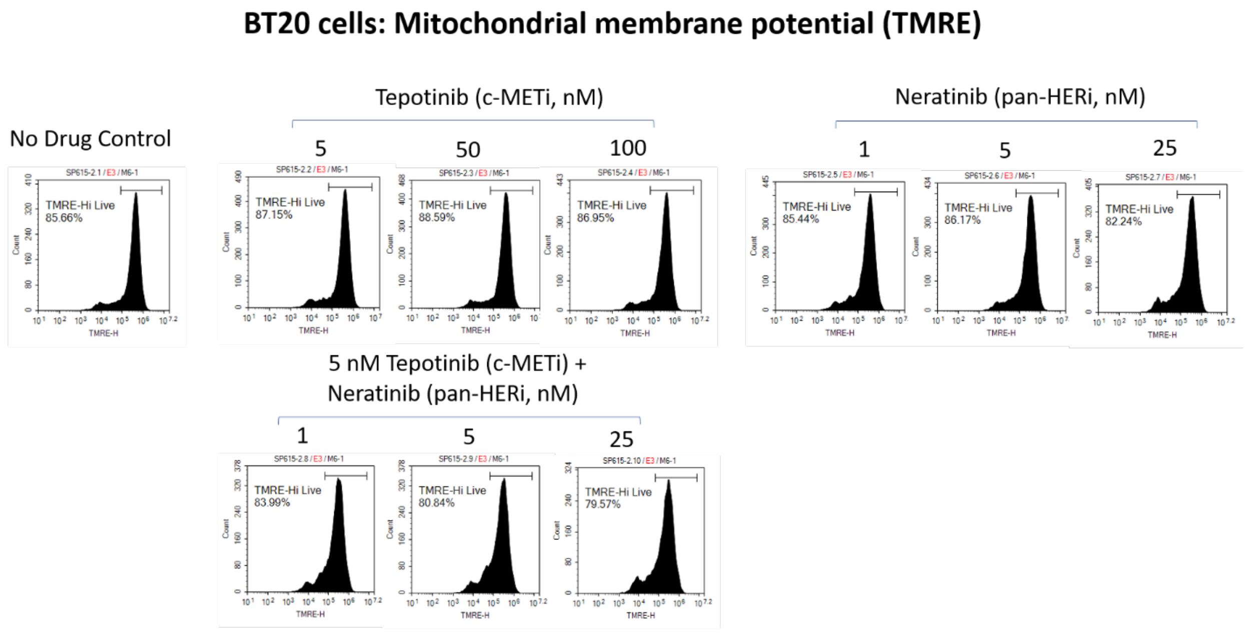

Fig. S5

Figure S11. CELsignia test is more sensitive and rapid for detecting anti-cancer drug efficacy compared to biological correlates – Normal signaling cell line, BT-20 continued. BT-20 cells were seeded in collagen-fibrinogen (CF) coated culture plates and treated 6h later with tepotinib (250 nM) and neratinib (250 nM), either singly or in combination, as indicated. Cells were harvested and then analyzed by flow cytometry for the markers shown following a period of 16h drug treatment. BT-20 cells do not respond in this timeframe to these drugs according to TMRE marker.

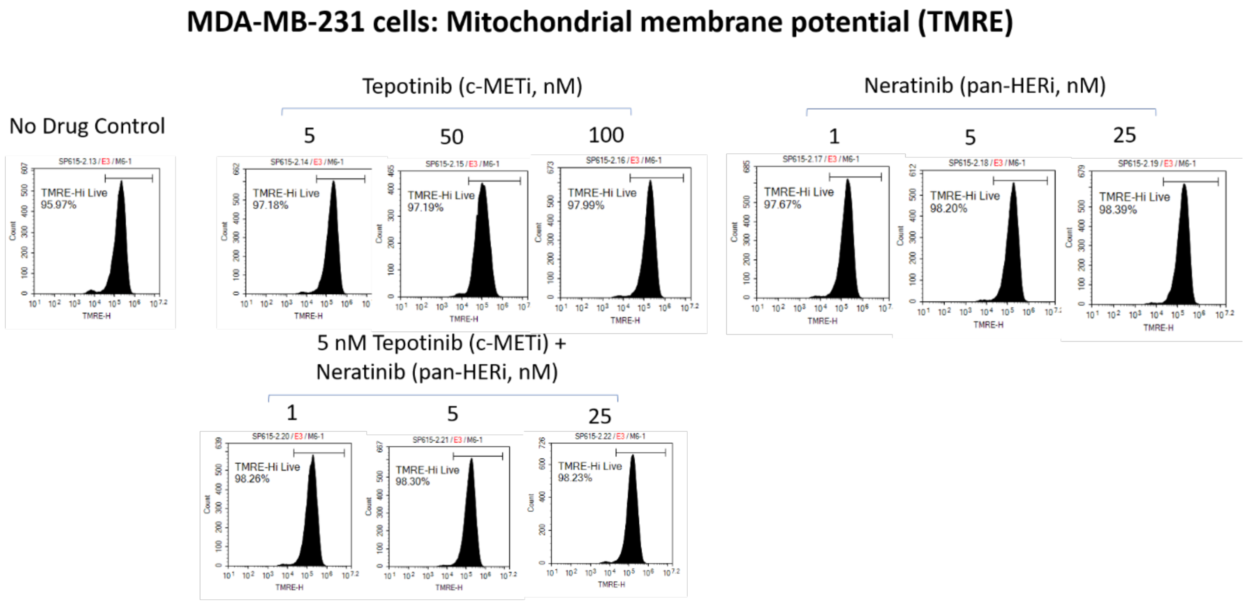

Fig. S6

Figure S12. CELsignia test is more sensitive and rapid for detecting anti-cancer drug efficacy compared to biological correlates – Normal signaling cell line, MDA-MB-231 continued. MDA-MB-231 cells were seeded in collagen-fibrinogen (CF) coated culture plates and treated 6h later with tepotinib (250 nM) and neratinib (250 nM), either singly or in combination, as indicated. Cells were harvested and then analyzed by flow cytometry for the markers shown following a period of 16h drug treatment. MDA-MB-231 cells do not respond in this timeframe to these drugs according to TMRE marker.

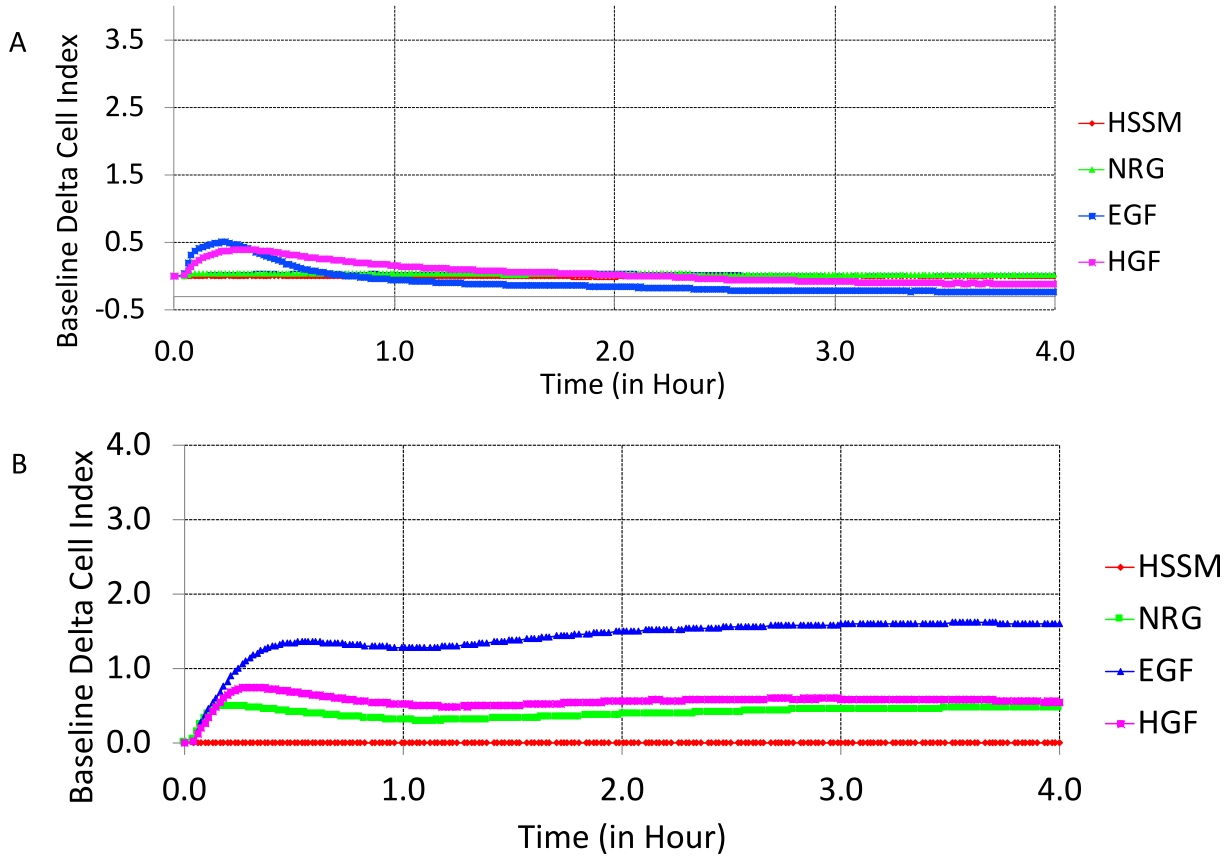

S13: Impedance time course of growth factor stimulation for two breast cancer cell lines that display a normal CELsignia HER2 family score and a normal CELsignia c-MET score (<250). NRG, EGF and c-MET were added individually as agonists A) MDA-MB-231 B) BT20 cell lines along with cells alone (HSSM, red line).

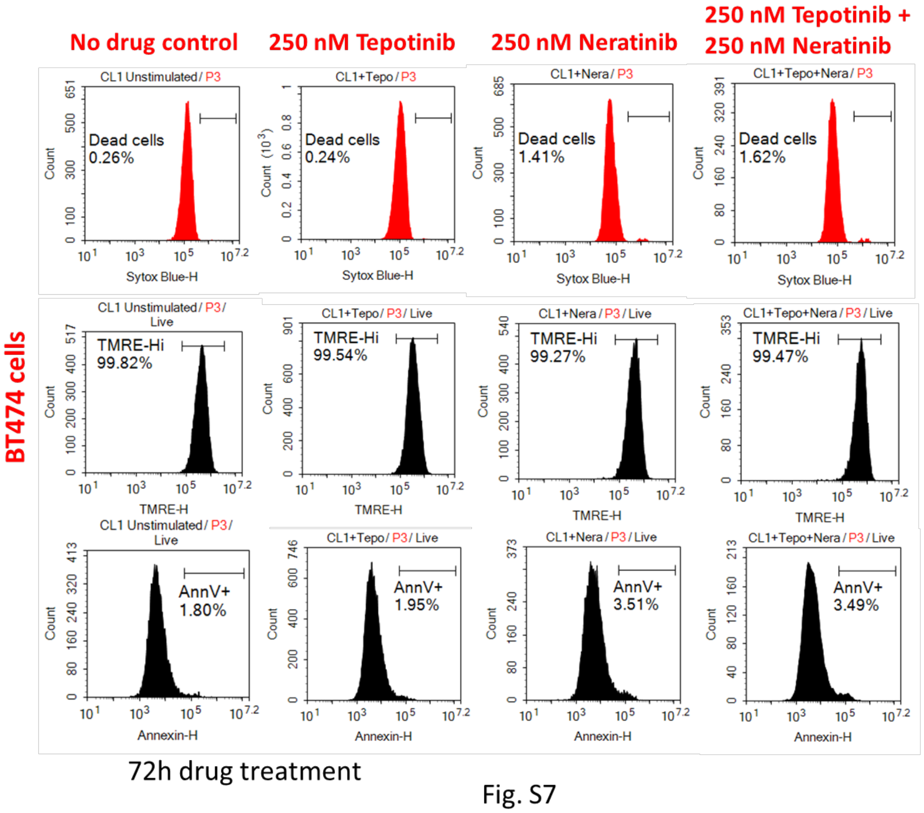

Fig. S7

Figure S14. Cell lines sensitive by functional real-time CELsignia tests exhibit cytotoxicity marker resistance to prolonged drug treatment – Normal signaling cell line, BT474. BT-474 demonstrated normal signaling by the CELsignia test. These cell lines were treated with neratinib and/or tepotinib for 72h at 6h post-cell seeding as described in Figure 6 and then analyzed by flow cytometry for cell death using Sytox Blue, apoptosis marker annexin V, and mitochondrial potential by TMRE staining. Flow cytometry histogram plots are representative of at least two independent experiments. BT-474 cells do not respond to these drugs under protracted conditions according to these markers.

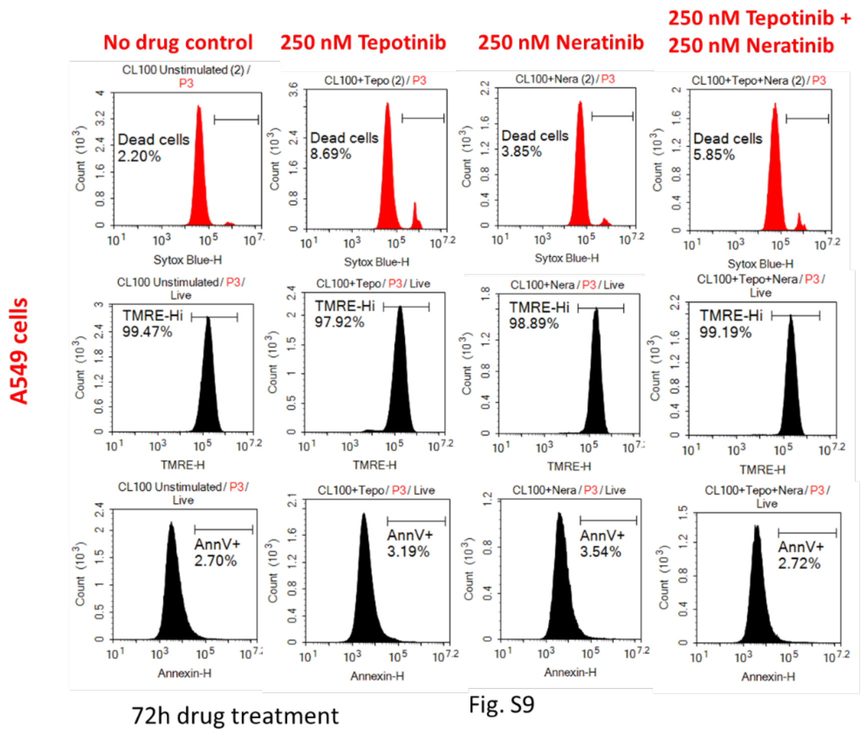

Figure S15. Cell lines sensitive by functional real-time CELsignia tests exhibit cytotoxicity marker resistance to prolonged drug treatment – Normal signaling cell line, A549. A549 cell demonstrated normal signaling by the CELsignia test. A549 cells were treated with neratinib and/or tepotinib for 72h at 6h post-cell seeding as described in Figure 6 and then analyzed by flow cytometry for cell death using Sytox Blue, apoptosis marker annexin V, and mitochondrial potential by TMRE staining. Flow cytometry histogram plots are representative of at least two independent experiments. A549 cells do not respond to these drugs under protracted conditions according to these markers.

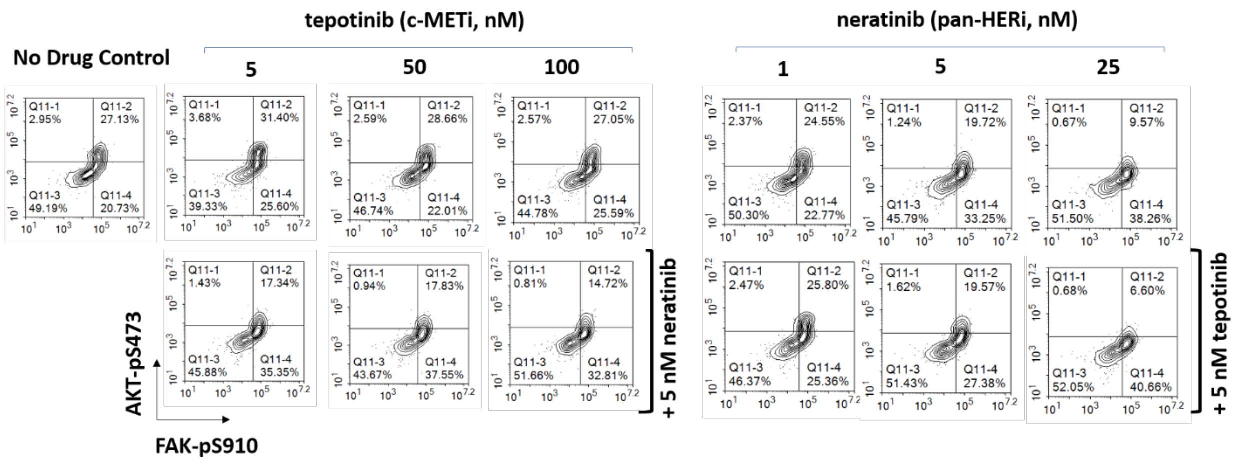

Fig. S11

Figure S16. pFAK^{high}pAKT^{low} change is found co-expressed in a significant population of pan-HERi or pan-HERi+c-Meti treated cells leading to apoptosis. HCC1954 cells were treated and analyzed as described in Figure 7. This figure shows the biological replicate data from tests performed the same day for the Figures 7A-B in the main text.

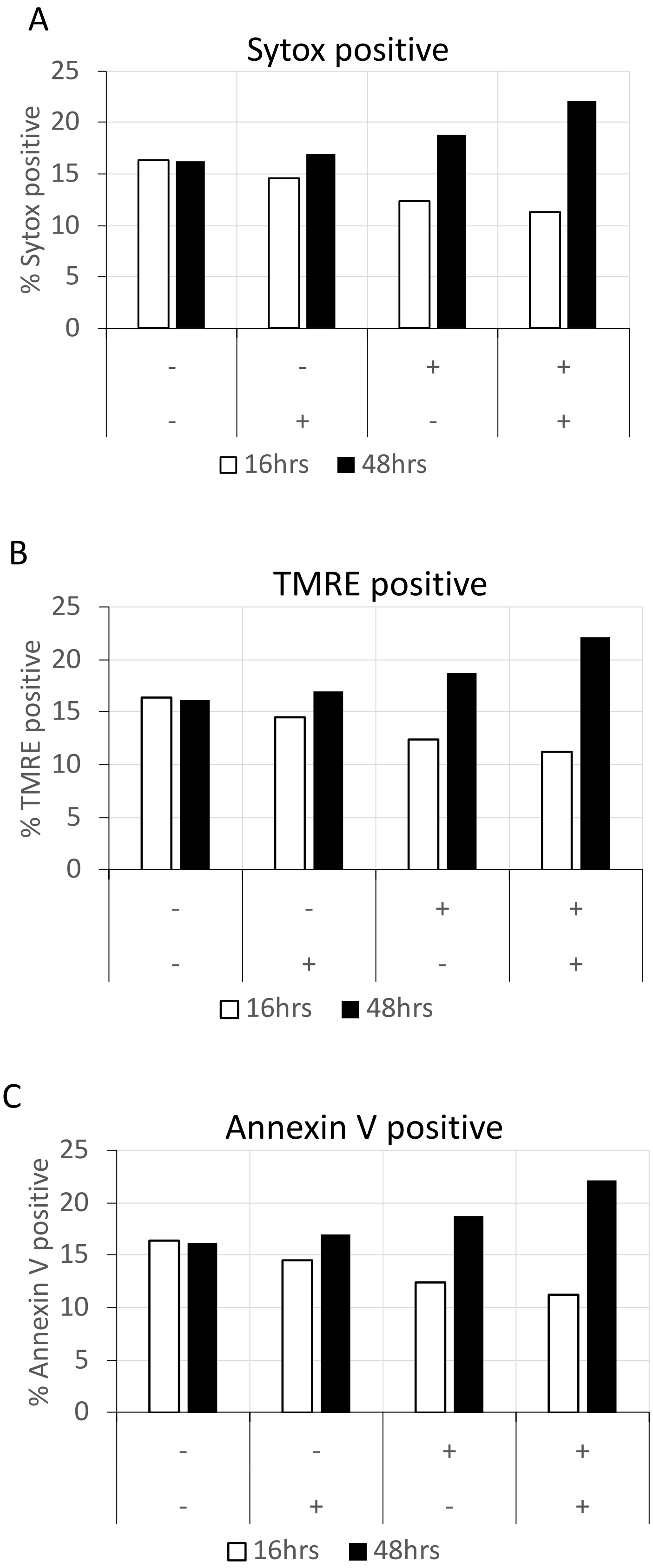
A
Sytox positive
25
20
15
10
5
0
% Sytox positive
- - + +
- + - +
16hrs
48hrs
B
TMRE positive
25
20
15
10
5
0
% TMRE positive
- - + +
- + - +
16hrs
48hrs
C
Annexin V positive
25
20
15
10
5
0
% Annexin V positive
- - + +
- + - +
16hrs
48hrs

Figure S17. Tabular data taken from Figure 6 represented as vertical bar graphs. HCC1954 cells treated for 16 or 48 hours with neratinib or tepotinib and analyzed by flow cytometry. A) percent sytox positive as a measure of membrane permeability, B) percent TMRE positive as a measure of mitochondrial health and C) percent Annexin V positive as a measure of apoptosis.

Table S1. Patient characteristics based on age, stage of cancer, tumor histology, and expression of estrogen receptor in tumor cells. All patients enrolled in this study expressed normal levels of HER2 receptor.

Characteristic	Number of Patients	%
Total Patients	79	100%
Age, years		
Mean	56.4	
Range	34-87	
Clinical Stage		
I	12	15%
II	49	62%
III	15	19%
IV	3	4%
Histology		
DCIS only	1	1%
Invasive only	14	18%
Invasive Ductal/DCIS mixed	45	57%
Lobular/other	19	24%
Lymph Status		
Positive	40	46%
Negative	36	51%
pNx or N/A	3	4%
Estrogen Receptor Status		
ER+	69	87%
ER-	10	13%

Table S2. Antibodies used in the study

Antigen	Conjugate	Clone	Source
CD49f	PerCP-eFluor710	eBioGoH3	eBioScience
EPCAM	AlexaFluor488	MH99	eBioScience
HER2	Phycoerythrin	24D2	Biolegend
HER3	PerCP-eFluor710	SGP1	ThermoFisher
c-MET	AlexaFluor488	95106	R&D Systems
FAK-pS910	AlexaFluor488	558544	BD Biosciences
AKT-pS473	AlexaFluor647	4075	Cell Signaling Technology

Table S3: Selected dose combinations from median effects analysis for 3 patient-derived cells demonstrating growth factor combinations leading to antagonism

Patient ID	EGF (pM)	NRG(pM)	HGF (pM)	Median Effect	C_{index}
C753	12	0	2	0.344	8.4
C753	2.4	0	0.4	0.14	3.8
C753	2.4	24	0	0.229	3.64
C753	0	24	0.4	0.176	3.02
C899	12	0	2	0.301	5.93
C899	2.4	0	0.4	0.117	2.42
C899	2.4	24	0	0.263	0.95
C899	0	24	0.4	0.231	1.07
C1061	12	0	2	0.597	2.65
C1061	2.4	0	0.4	0.242	1.08
C1061	2.4	24	0	0.432	0.825
C1061	0	24	0.4	0.067	1.85
